# Quantifying the Spatial and Temporal Distributions of Volatile Chemical Products (VCPs) in the Greater Houston Area

**DOI:** 10.1021/acs.est.4c13855

**Published:** 2025-06-26

**Authors:** Alana J. Dodero, Sining Niu, Heewon Yim, Kyle P. McCary, Sahir Gagan, Yeaseul Kim, Timothy B. Onasch, James H. Flynn, Raghu Betha, Karsten Baumann, Sarah D. Brooks, Qi Ying, Yue Zhang

**Affiliations:** † Department of Atmospheric Sciences, 14736 Texas A&M University, College Station, Texas 77843, United States; ‡ Department of Civil and Environmental Engineering, Texas A&M University, College Station, Texas 77843, United States; § 53777 Aerodyne Research, Billerica, Massachusetts 01821, United States; ∥ Institute for Climate and Atmospheric Science, 14743 University of Houston, Houston, Texas 77204, United States; ⊥ Department of Civil, Environmental, &; Construction Engineering, 6177 Texas Tech University, Lubbock, Texas 79409, United States; # Picarro, Inc., Santa Clara, California 95054, United States; 7 Department of Environmental Sciences and Engineering, 2331 University of North Carolina, Chapel Hill, North Carolina 27599, United States

**Keywords:** Volatile chemical products, mobile measurements, anthropogenic emissions, Vocus 2R CIMS

## Abstract

Volatile chemical products (VCPs), including organic species emitted from pesticides, coatings, cleaning products, and personal care products, account for more than half of the urban VOC emissions in major North American and European cities. However, VCP emissions, spatial and temporal distributions, and impacts vary widely. Despite being the fourth largest U.S. city, Houston, Texas, lacks measured VCP concentration and emission data. This study presents the first spatial and temporal measurements of selected VCP tracers in Houston, Texas, using a Vocus 2R Chemical Ionization Mass Spectrometer on a mobile platform. Ambient measurements of five major VCP tracers, including D5-siloxane, monoterpenes, *para*-dichlorobenzene, *para*-chlorobenzotrifluoride (PCBTF), and 2,2,4-trimethyl-1,3-pentanediol isobutyrate (Texanol), were collected in winter and summer 2023. Several compounds exhibited significantly higher averaged concentrations, with pronounced spatial and seasonal variability, distinguishing Houston from urban areas in the temperate and cooler climate zone. A customized box model was employed to estimate seasonal emissions for the Greater Houston Area, showing that emissions of most VCPs were significantly higher during the summer. This study provides critical insights into the distribution and emission of VCPs in a subtropical metropolitan area, advancing methods for assessing VCP emissions and concentrations across cities and improving understandings of their impacts on air quality, climate, and public health.

## Graphical abstract

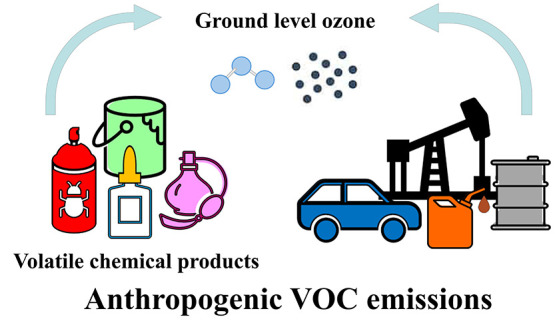

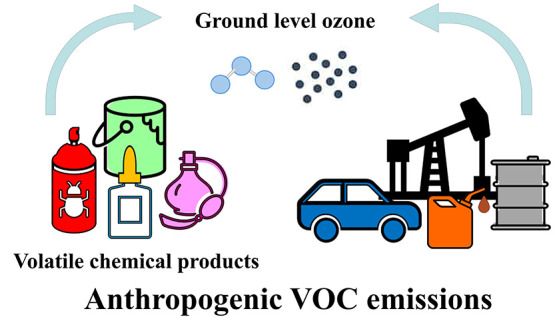

## 1 Introduction

Air pollution is the world’s largest environmental cause of disease and premature death,^1,2^ with volatile organic compounds (VOCs) serving as a significant contributor.^3–5^ VOCs can undergo atmospheric oxidation to produce major atmospheric pollutants such as photochemical ozone (O_3_) and secondary organic aerosols (SOA),^6–10^ which have adverse health effects on the general population.^1,11–15^ In urban areas, anthropogenic VOCs dominate the total VOC emissions and had been traditionally linked to traffic and tailpipe emissions, power plants, and residential combustion.^16–22^ However, traffic-related VOCs have significantly decreased in the past few decades due to regulations and policies,^23–26^ increasing the relative contribution of other VOCs such as volatile chemical products (VCPs).^6,22,27^

VCPs typically consist of organic species found in cleaning agents, printing inks, personal care products, pesticides, and coatings.^6,27–29^ However, the emission, spatial and temporal distribution, and impacts of VCPs remain largely uncertain.^30–32^ For instance, VCP emissions strongly depend on population,^27,28^ climate,^33^ and the number and type of consumer and business activities in an area.^28^ Therefore, there is a significant variation in the spatial and temporal distributions of VCPs across different cities,^34,35^ highlighting the need for highly resolved temporal and spatial VCP measurements, especially in major urban areas with diverse characteristics. Recent studies conducted in New York City, Los Angeles, and a few European cities have shown that VCPs already account for half of the VOCs in urban areas,^6^ with an even increasing trend as tailpipe emissions continue to decline.^27,28^ Such results may profoundly impact future air quality, climate, and public health.^25^ However, most of the previous VCP studies were conducted in the temperate climate zone with cooler temperatures.^6,27^ Currently, there are no reported VCP concentrations and emissions in subtropical climate zones comparable to those of cities in the Southern U.S. or Southern Europe. Differences in temperature and humidity for cities in the subtropical regions may contribute to different VCP emission rates, potentially affecting their transformation rates and lifetimes.^36,37^ This leads to uncertainties in quantifying VCP emissions and their impacts on air quality and climate. Additionally, the seasonal trend of VCPs is largely unknown.^6,38^

As the second largest state in the United States with regard to population, Texas has several major metropolitan areas representative of cities in the subtropical region. The National Emissions Inventory (NEI) demonstrates that VOC emissions in Texas are VCP-dominated, similar to those in Los Angeles and New York City. However, the actual VCP contribution to the VOC emissions are not well understood as NEI may underestimate VCP emissions.^39^ For example, McDonald et al. demonstrated that VCP emissions in Los Angeles may be higher than the NEI by a factor of 2–3.^27^ The US EPA developed the VCPy emission inventory using a new modeling framework,^22^ showing VCP emissions in Texas to be 247 Gg, higher than the NEI estimation of 212 Gg in 2017.^22^ Discrepancies in emission estimates from the above emission inventories highlight the need for field measurements to constrain uncertainties in model parametrization. Being the largest city in Texas and the fourth largest city in the U.S., Houston has more than 7 million people in the surrounding areas,^40^ serving as an ideal example for examining the spatial and temporal distributions of VCPs for cities in the subtropical region.

Therefore, this study measured major tracer compounds for VCPs, in the Houston area using a Vocus 2R CIMS, being the first to report the spatial and temporal concentrations and emissions as well as seasonal variations of VCPs in a subtropical city. We specifically focus on 5 major VCP tracers identified by Gkatzelis et al.,^6^ including D5-siloxane from personal care products, monoterpenes from fragrances, *para*-dichlorobenzene from insecticides, Texanol from water-based coatings, and PCBTF from solvent-based coatings.^41^ A box model was constructed to estimate the emission profiles of these major VCP species in the Greater Houston Area (GHA) and compare such data with emissions from temperate and cooler regions.

## 2 Materials and Methods

### 2.1 Field Deployment and Instrumentation

Field measurements of VCPs in the GHA were conducted using the Vocus 2R CIMS on a mobile platform. Specifically, the Texas A&M University (TAMU) Rapid Onsite Atmospheric Measurement Van (ROAM-V) was deployed to measure the urban atmospheric composition across the GHA. The ROAM-V is equipped with a Vocus 2R CIMS, a relative humidity (RH) and temperature sensor, a GPS monitor, and other gas- and particle-phase measurement instruments. Figure S1 in the Supporting Information (SI) shows a schematic of the van layout, including additional instruments that were not used in this work. More information regarding the van specifications can be found in Section S1.1 of the SI.

Mobile measurements were conducted in Houston from January 17–24th and August 10–26th, 2023. Measurement dates and times along with average meteorological conditions for each route are summarized in Table S1 of the SI. Measurements were made on two different routes to obtain comprehensive spatial distributions of VCPs around the Houston area, encompassing both high- and low-population areas. These routes for winter and summer are shown in Figure S2 of the SI. Although the winter and summer routes differ slightly, both include a round route, encircling Downtown Houston and a North–south route. Each route began at the University of Houston (29.72° N, 95.34° W). The circular route traversed the downtown area and circumnavigated the major metro area within Highway 99. The North–south route passed through the highly populated metro area and to areas of lower population to the North and South, moving parallel to Highway 288 and Interstate 45. Major highways and interstates were avoided whenever possible to minimize the influence of tailpipe emissions on the measurements. The instruments were continuously monitored from within the van during each sampling drive to ensure proper performance. GPS coordinates were recorded every second using a VK172 G-Mouse USB GPS receiver for Windows. Temperature and relative humidity were measured every second using an OMEGA RH sensor.

Time series data on chemical composition and mass spectra were collected with the Vocus 2R CIMS. The Vocus 2R CIMS ionizes the gaseous analyte via gas-phase ion–molecule reactions.^41^ In this study, the Vocus 2R CIMS was deployed with proton transfer reaction mode (PTR mode),^42^ using H_3_O^+^ as the reagent ion.^41,43,44^ More information regarding the working principles of the Vocus 2R CIMS is provided in Section S1.2 of the SI. Ambient air was drawn into a 3-m-high inlet on top of the van at a flow rate of 5 L/min (lpm) through 3/8 in. polytetrafluoroethylene (PTFE) tubing to reduce the residence time before reaching the instrument. Ambient air entered the Vocus 2R CIMS at a flow rate of 100 standard cubic centimeters per minute (sccm), and the remaining air passed through a bypass pump. This instrument was operated between 20 and 30 °C, and the relative humidity of the sample was controlled to be less than 60% by insulating the gas inlet with slightly heated Styrofoam sleeves. The Vocus 2R CIMS is not sensitive to RH in the PTR mode.^45^ Therefore, the air was not dried before entering the Vocus 2R CIMS. Mass spectra were collected every second and averaged at 10-s intervals before processing using the Tofware package in Igor Pro (WaveMetrics).^46^ Time series were examined to identify outliers, and outliers exceeding three times the standard deviation for each day were removed from the 10-s data. This approach did not eliminate plumes from the data set; rather, it filtered out data points that lacked notable trends and were likely caused by instrument noise. The Vocus 2R CIMS data collected during the winter and summer periods were analyzed to characterize seasonal and spatial differences of VCPs in the ambient environment in the GHA. These VCPs include monoterpenes from fragrances, D5-siloxane from personal care products, *para*-dichlorobenzene from insecticides, parachlorobenzotrifluoride (PCBTF) from solvent-based coating emissions, and Texanol from water-based coatings and adhesives. Table S2 in the SI lists the VCPs included in this study, and Figure S3 in the SI shows the peak fittings for each compound. Although these tracer VCP compounds represent only a subset of total VCPs, they were selected for their ability to characterize different sources and facilitate comparisons to previous studies. Additional compounds, such as other volatile methyl siloxanes and aromatic solvents, can also be used as tracers for VCPs.^47,48^ Similarly, monoterpenes have been used as a tracer for fragrances,^6^ but it is important to note that monoterpenes also have significant biogenic sources.^49^ Considering this study only focused on tracer VCPs and noncombustion related compounds, corrections for the van exhaust were not performed.^6^

### 2.2 Calibration Procedure

Calibration curves were created after each field campaign for α-pinene, D5-siloxane, *para*-dichlorobenzene, PCBTF, and Texanol from VCP standards (Apel-Riemer Environmental, Inc. Miami, Florida) to determine their respective detection limits and sensitivities in the Vocus 2R CIMS. Initially, zero air from an ultrahigh purity zero air cylinder (Airgas, Inc.) was injected through a solenoid valve at a flow rate of 300 sccm for five min to establish a clean instrument background. The calibration gas, containing 1000 ppb of each VCP standard mixed with nitrogen gas, was then injected at flow rates of 1, 2, 3, 4, and 5 sccm for five min each, achieving concentrations of 3.3, 6.6, 9.9, 13.2, and 16.4 ppb, respectively. Calibration curves for each compound were established by performing a linear least-squares regression of the signal of each species from the Vocus 2R CIMS as a function of their concentration from the calibration gas (Figure S4 in the SI). The ratio of the sensitivity of each compound to the sensitivity of α-pinene was used to calculate the concentrations for each VCP. Zero air signals were obtained for three min every hour during mobile measurements and for 5 min after mobile sampling. The period with the lowest average was subtracted from the data for each measurement day. The limit of detection (LOD) for each compound was determined as three times the standard deviation of the zero air signals for each day multiplied by the sensitivity between each compound and α-pinene. Data points below the LOD were removed. Since all tracer VCPs were above their respective LODs, this removal did not introduce a positive bias but simply eliminated negative or low values resulting from instrument noise.

Vocus 2R CIMS is sensitive to changes in the environment.^50^ Therefore, hourly Vocus 2R CIMS sensitivity calibrations were performed during the field campaign with a calibration gas cylinder containing the 5 major VCPs analyzed in this study (Apel-Riemer Environmental, Inc. Miami, Florida). During calibration, zero air was injected at a flow rate of 300 sccm for 3 min. Then, the calibration gas was injected at a flow rate of 5 sccm for two min, leading to a concentration of α-pinene of 16.4 ppb. The Vocus 2R CIMS measured the diluted calibrants, and the calibration factor for α-pinene was derived in units of ions/second/ppb (Figure S5 in the SI). Hourly calibrations were individually analyzed to select periods of stable concentration during the calibration, and the average concentration was taken for each stable period. Linear interpolations between hourly calibrations were used to correct the data between each calibration point (Figure S5 in the SI). Considering that the calibration gas was introduced through a solenoid valve in the Vocus 2R CIMS instead of through the inlet, tubing loss experiments were conducted to assess potential losses within the inlet line. Figure S6 of the SI presents the tubing loss experiments for tubing lengths of 1, 2, and 3 m. These experiments indicate that no significant losses occurred between the van’s external inlet and the instrument.

### 2.3 VCP Concentration

The ambient VCP concentrations were calculated based on the calibration curves and signal obtained by the Vocus 2R CIMS. Since the ROAM-V sampled VCPs at different times of day and locations, the planetary boundary layer height (PBL) also plays an important role in affecting the VCP concentration. To account for environmental changes throughout the day and provide uniform metrics for VCP concentrations, normalized VCP concentrations (*C*_*norm*_) were calculated from ambient VCP concentrations (*C*) and divided by the PBL height using eq 1. *H*_*i*_ represents the PBL height at a given time, and *H*_*avg*_ represents the average PBL height for each day.

1 $$C_{norm}=C\times \frac{H_{i}}{H_{avg}}$$

A Weather Research & Forecasting (WRF) model was employed to provide hourly time series of the PBL height for each sampling day, averaged over the entire sampling domain. These hourly PBL heights were linearly interpolated to a 1 min resolution. VCP concentrations were averaged every minute and normalized with the PBL height. Wind direction and speed data collected from the nearby George Bush Intercontinental Airport (29.99° N, 95.34° W) in Northern Houston and William P. Hobby Airport (29.38° N, −95.16° W) in Southern Houston indicated relatively stable wind speeds throughout the sampling domain. Wind speeds averaged 6 ± 3 m/s during the winter and 3 ± 2 m/s during the summer (Table S1 in the SI).^51^ Similarly, the wind speed was relatively consistent between the two locations. Therefore, VCP concentrations were not normalized to the wind speed.

### 2.4 Emission Calculations

This study presents a method for calculating VOC emissions from either mobile or stationary measurements. Given that data was obtained across a wide area around Houston at varying times of day in multiple seasons, the average concentrations derived from the mobile measurements can provide an estimate of representative VCP concentrations for Houston as a whole. The error in the emission calculation also reflects the varying concentrations throughout the GHA. The upper and lower bounds of the calculated emissions serve as estimates for emissions from point sources and background areas, respectively. The upper bound reflects contributions from localized, high-emission sources, while the lower bound represents baseline emissions in areas with minimal direct source influence.

VCP emissions were calculated using a box model with a mass balance approach that assumes a uniform PBL height and mixing conditions throughout the Houston sampling area. This assumption is supported by the widely distributed population density (Figure S7 of the SI) and the negligible correlation between population density and VCP concentrations. Figure S7 of the SI shows that while Houston’s population density varies locally, with areas of both high and low concentrations, these variations are dispersed throughout the sampling domain. As a result, the domain can be reasonably treated as uniform. Figure S8 (see SI) shows the average PBL height during the summer sampling period and highlights when the ROAM-V measured the ship channel area. The daily emission rate for the 18,000 km^2^ area surrounding Houston is calculated using the following model and eq 2:

$$\frac{dC}{dt}=-k_{OH}\left[OH\right]\left[C\right]-k_{O_{3}}\left[O_{3}\right]\left[C\right]-\frac{v_{d}}{H}\left[C\right]+\frac{E}{H}$$ 2

Assuming $\frac{dC}{dt}=0$ , this equation can be rewritten in terms of emission rate to obtain eq 3:

3 $$E=H\left[C\right]\left(k_{OH}\left[OH\right]+k_{O_{3}}\left[O_{3}\right]\right)+v_{d}\left[C\right]$$

Here, *C* is the normalized average concentration for each day, *OH* is the daily averaged hydroxyl radical concentration, *O*_3_ is the daily averaged ozone concentration, *k*_*OH*_ and *k*_*O*_3__ are reaction rates of each VCP compound with OH· and O_3,_ respectively, *v*_*d*_ is the deposition rate for each compound, *H* is the daily averaged PBL height, and *E* is the emission rate. Emission rates were calculated using both normalized and non-normalized concentrations. OH· radical concentration, O_3_ concentration, and deposition rates were provided from the Community Multiscale Air Quality (CMAQ) model.^52–54^ The PBL height was obtained from the Weather and Research Forecasting Model (WRF) Version 4.5.1. More information regarding the CMAQ and WRF modeling can be found in Section S3.3 of the SI. Figures S9–S15 and Table S5 provide an overview of the model performance evaluations. Similarly, Figures S16–S18 provide the model outputs for the O_3_, OH·, and planetary boundary layer (PBL) height. Model simulations varied each day and were averaged over the entire measurement domain. Therefore, the box model provides an average emission over the sampling period and may have some uncertainties. Considering the meteorological conditions changed throughout the measurement period, with variable wind speed and direction, advection was not considered in the box-model. This method may lead to uncertainty in the emission estimate. Reaction rate constants for VCP reactions with hydroxyl radical and O_3_ were obtained from various laboratory studies and are summarized in Table S4 of the SI. Emission error was calculated using an error propagation method described in Section S3.1 of the SI (see equations S2 and S3). The per capita emission rate was determined by taking the average population in the sampling domain per square kilometer as obtained from WorldPop.^40^

### 2.5 Population Density

Correlations between VCP concentration and human population density were examined to determine if the VCP concentration depends on population density. Population density data for the Houston area was obtained from WorldPop.^40^ This data set provided a 1 km resolution population density for 2020. Although this is the most recent population density data, increases in population between 2020 and when this study was conducted may lead to an underestimation of population density in certain areas.^55^ Correlations between VCP concentration and population were obtained by comparing the population within a 1 km grid cell to the average VCP concentration within that grid cell. Correlations between population density and concentration were examined including non-normalized concentrations and concentrations normalized to the PBL height, benzene,^6,56^ and both.

## 3 Results and Discussion

### 3.1 Spatial Distribution

A total of 6 tracer VCP compounds identified by Gkatzelis et al.^6^ were analyzed in this study to provide comprehensive spatial and temporal distributions of major VCPs. Among these 6 compounds, D4-siloxane was below the LOD and is therefore not reported here. The detection limits of the remaining 5 VCP tracers were between 1 and 10 ppt in the Vocus 2R CIMS. Among these 5 detected VCP compounds, PCBTF and Texanol exhibited fragmentation in the Vocus 2R CIMS operating in the H_3_O^+^ mode.^6^ Therefore, time series data for their respective fragment ions are reported. The fragments or molecular ions of each VCPs were observed consistently on all deployment days.

Most of the measured VCPs show higher concentrations in the major metropolitan area within the Highway 99 loop, which encircles Houston (Figure 1 and SI Figure S19). Concentrations decreased further south in more rural areas. Additionally, elevated VCP concentrations were observed near the Houston Ship Channel, the busiest waterway in the United States, which transports over 230 million tons of cargo annually (see Figure S2 in the SI).^57^ The elevated concentrations of industrial VCPs such as PCBTF and Texanol in the ship channel could be due to transporting and producing goods containing these products along with activities associated with the industrial area.^6,58^ However, it is important to note that measurements were taken around the Houston Ship Channel in the early morning when the boundary layer was low. This contributes to the enhancement of the observed concentrations compared with other areas sampled later in the day. Figures 1–2, SI Figures S19–S20 (see the SI), and Table S3 (see the SI) show the comparison between the time-series and normalized VCP concentrations accounting for the influence of the PBL height variations during the day. The normalized concentrations for Texanol also show average or above-average concentrations around the ship channel, suggesting that the emissions of VCPs from this area are relatively higher than those from the rest of the city.

**Figure 1:**
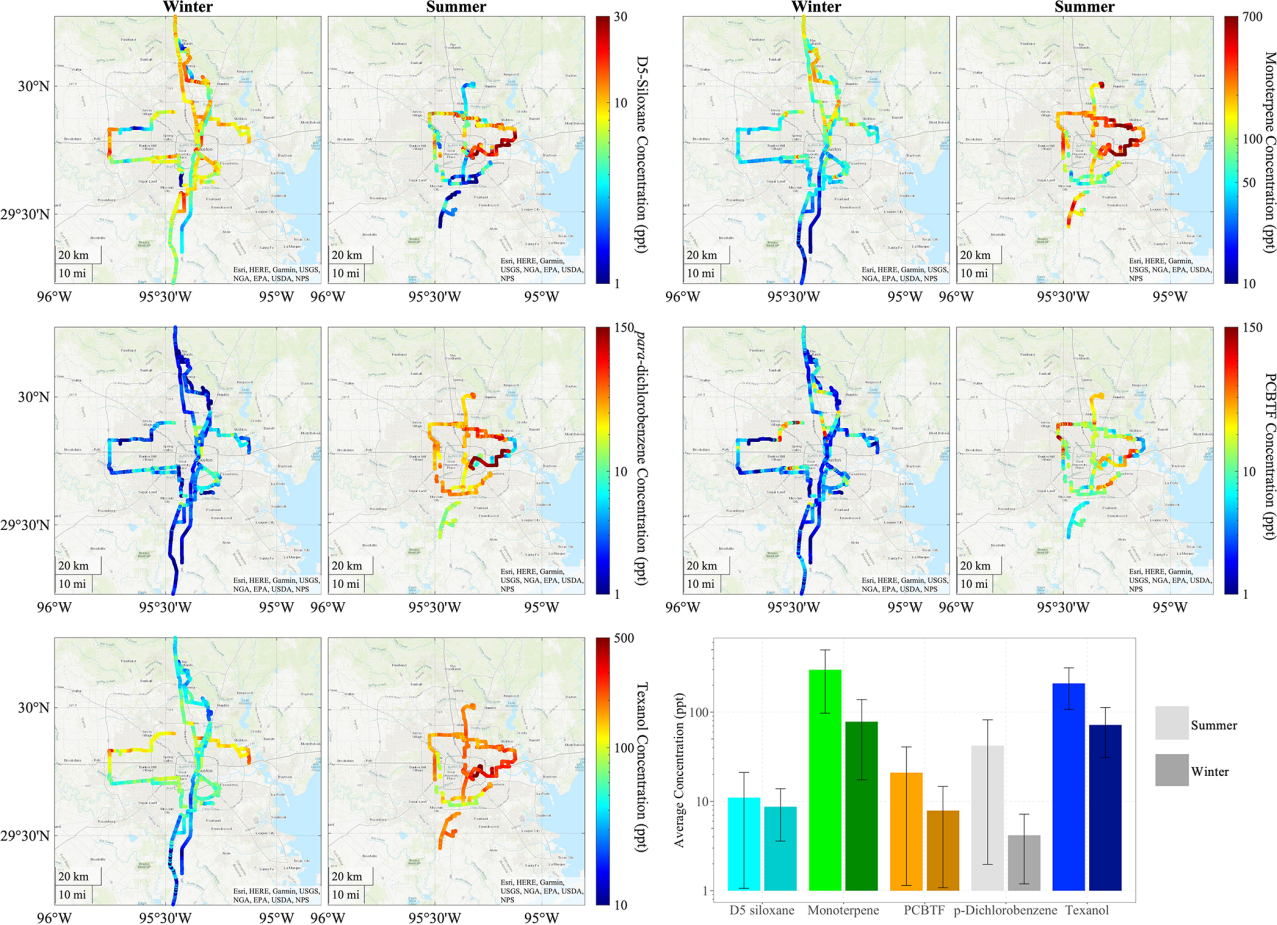
Spatial distribution of VCPs measured by the mobile laboratory, including D5-siloxane, Monoterpene, *p* *ara*-dichlorobenzene, PCBTF, and Texanol, for winter and summer. Note that the scales vary between compounds.

**Figure 2:**
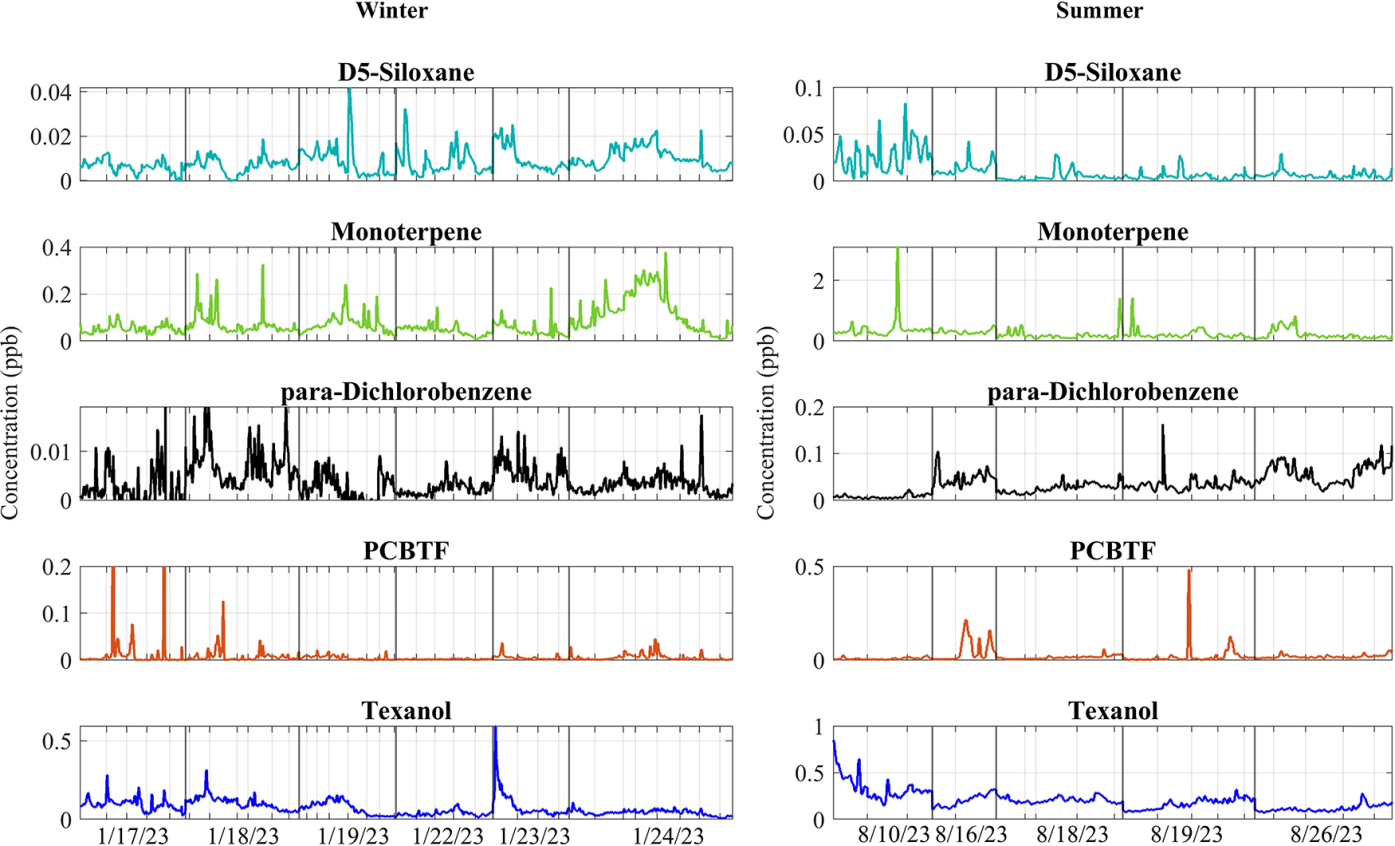
Normalized VCP time series of major VCPs, including D5-siloxane, Monoterpene, *para*-dichlorobenzene, PCBTF, and Texanol, for winter and summer. Note that the scales vary between seasons and compounds.

### 3.2 Concentration and Population Density

Houston’s population density is distributed within a 30 km radius of the city center, largely within the boundaries of Highway 99, resulting in a more dispersed population density across the metropolitan area, as shown in Figure S7 (see the SI). Other major cities, such as New York City, have distinct areas of high population density near the city center and then steadily decreasing population further out.^6,28,59^ To determine whether population density impacts VCP concentrations, correlations between population density and each VCP concentration were examined for each sampling day. However, for most routes, no significant correlation was found between VCP concentration and population density at the 5% significance level, regardless of whether concentrations were non-normalized or normalized to the PBL height and benzene. Given the high concentrations of VCPs observed in the Ship Channel despite its lower population density, population correlations were also examined, both including and excluding the Ship Channel area. However, no significant correlation was found for any of these methods. This lack of correlation is likely due to the PBL being well-mixed during most of the drive routes and the point sources of VCPs being evenly distributed throughout Houston. August 18th was the only day with a notable correlation between D5-siloxane and population density (R^2^ = 0.5). This correlation was likely influenced by the North–south transect of the sampling route, which passed through regions with changes in population density, from rural areas in the South to densely populated areas in the middle and North. Additionally, the wind was mainly coming from the Southeast on this day, which may have limited the transport of VCPs from the Houston metropolitan area to the less populated areas South of Houston. On the other sampling days, winds shifted often, bringing air in from multiple directions.

The lack of correlation could be due to a wide distribution of population density throughout the sampling domain and the transport of VCP species away from their sources. Many of the VCPs studied in this work have atmospheric lifetimes of multiple days. For example, D5-siloxane, monoterpene, *para*-dichlorobenzene, and PCBTF have lifetimes of about 4, 1, 36, 1, and 48 days, respectively.^6^ Therefore, they can either accumulate in the air or be transported far away from their source region. Alternatively, monoterpenes and Texanol have shorter lifetimes of less than a day.^6^ However, monoterpenes and Texanol are tied to biogenic and industrial sources, respectively, which may not correspond to areas with a high population. Additionally, the lack of correlation may be attributed to the limited sample size obtained from the mobile data. For instance, the van may only briefly traverse the 1 km grid used to determine population density, resulting in insufficient data representation. Likewise, the time of day at which the measurements are conducted could also influence the observed lack of correlation.

### 3.3 VCP Emissions and Concentration

VCP emissions were calculated by using a mass balance approach. Emission rates were obtained in mg person^–1^ day^–1^, assuming a uniform PBL height, concentrations of VCP, ozone, OH·, and population throughout Houston. Eq 3 assumes uniform concentration and emissions for the entire day and, therefore, represents average daily emissions. Both emissions and concentrations of VCPs have a diurnal profile, with enhancement during the middle of the day and low values at night. Therefore, the mean concentration from each sampling day was used to calculate the emission from 6:00–23:00, and the background concentration was used to calculate the emission from 23:00–6:00. The background concentrations were determined by taking the average of the period with the lowest concentrations for each day with the goal of better representing the lower emissions of VCPs during the night.

All VCP species exhibited higher emissions in summer compared with winter, primarily due to increased observed concentrations. Higher VCP, ozone, and OH· concentrations associated with warmer temperatures and more intense sunlight along with elevated boundary layer heights further contribute to the seasonal difference. Greater emissions are required in the summer to reach the measured concentrations, reflecting the combined effects of atmospheric chemistry and meteorological conditions. D5-siloxane and monoterpenes had the highest derived emissions, similar to what was reported in Gkatzelis et al.^6^ Emission values are summarized in Figure 3 as well as Tables S6 and S8 of the SI. To assess the impact of daily variations in the PBL height on emission estimates, emissions were calculated using both average concentrations and normalized VCP concentrations. A comparison of these methods is presented in Table S6 of the SI. While the overall differences between the two approaches were not significant, emissions derived from normalized concentrations were consistently lower.

D5-siloxane is commonly used as a tracer for personal care products. It is common in many cosmetics, such as deodorants, sunblocks, and skin care products, and has a relatively long lifetime. Higher concentrations of D5-siloxane were observed near residential areas and on highways, both of which can be linked to the use of D5-siloxane in personal care products. Coggon et al.^56^ demonstrate that spikes in D5-siloxane correlate to common vehicle emission tracers, such as benzene. This correlation is attributed to passengers in vehicles emitting D5-siloxane into the car cabin air, which is subsequently released from the vehicle.

**Figure 3:**
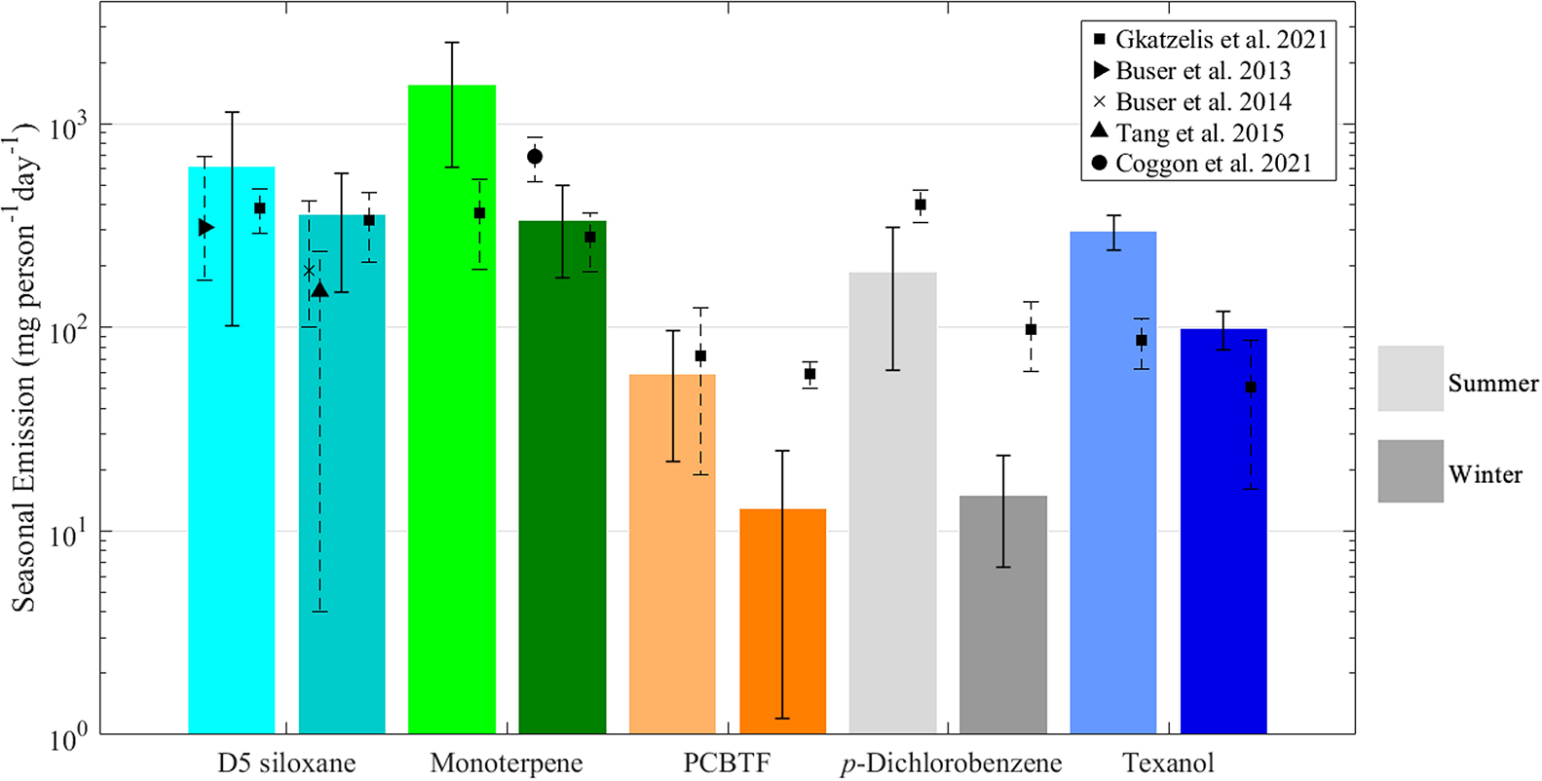
Emission values of VCPs from summer and winter seasons derived from the box model. Emissions for summer and winter are shown on the left in light colors and on the right in darker colors, respectively. Comparisons with previous studies are shown in different marker styles.

The mean concentrations of D5-siloxane in the winter and summer were 8.5 ± 5.2 and 10.1 ± 11.3 ppt (v/v). Such values are higher than values reported in Toronto, Canada^60^ and Boulder, USA^56^ but lower than those reported in New York City^6^ (see Table S7 in the SI). This difference is likely due to the population of Houston being larger than both Boulder and Toronto but smaller than New York City. The mean concentrations in the winter and summer were not significantly different, likely due to the continuous use of products containing D5-siloxane throughout the year. The emissions for D5-siloxane were estimated to be 358 and 620 mg person^–1^ day^–1^ for winter and summer, respectively, using the box model described in Section 2.4. These wintertime emission values for D5-siloxane are similar to those in Zurich, Switzerland^61^ and New York City^6^ but roughly two times higher than in Chicago.^62^ The summertime emissions are about twice as high as those reported in New York City likely due to higher temperatures and evaporation in Houston. Notably, Houston’s wintertime emissions are similar to those found in an indoor classroom environment.^48^ This similarity could potentially be due to concentrations accumulating in ambient air due to limited mixing. Similarly, summertime concentrations may be higher in this study compared to indoors due to higher temperatures and more evaporation of D5-siloxane. Note that emissions for Zurich were determined by wintertime measurements, Chicago from summertime, and New York City from both winter and summertime as seen in Table S8 of the SI.

Monoterpenes are primarily emitted from biogenic sources^49^ and are also associated with cooking and wood burning. However, Coggon et al.^28^ showed that monoterpene emissions are dominated by fragranced VCPs in urban areas in the temperate region. The mean concentration of monoterpene in the winter was 74.9 ± 59.6 ppt, which is similar to concentrations in New York City.^28^ It is worth noting that the winter measurements took place after a prolonged period of subzero freezing temperatures;^63^ therefore, biogenic sources were likely not significantly contributing to the wintertime concentrations. However, the summertime concentration was 249.6 ± 219.0 ppt, 75% higher as that of New York City,^6^ likely due to more biogenic sources in Houston’s subtropical environment and more evaporation of monoterpenes from personal care products. Such results are further supported by Figure 1, which shows the concentrations in the south of the sampling area during the summer as high as in the downtown area due to the South Houston area being rural with abundant vegetation. In contrast, the downtown areas are urban with more human activities and less vegetation; therefore, the summertime monoterpenes measured downtown are likely a mix of both biogenic and anthropogenic monoterpene sources. Emissions of monoterpenes were 334 and 1561 mg person^–1^ day^–1^ for the winter and summer, respectively. Coggon et al. 2021 estimated that anthropogenic monoterpene emissions in New York City during the winter range from 520 to 860 mg person^–1^ day^–1^ and that monoterpenes from fragranced VCPs were emitted at about 220 mg person^–1^ day^–1^.^28^ The emission estimate for Houston is also lower in the winter compared with New York City. Considering that Vocus 2R CIMS cannot differentiate between monoterpene isomers, the emission estimates reported here account for all monoterpene emissions. Summertime emissions were about four times higher than those in wintertime, which differs from previous studies.^6,28^ This difference may be attributed to higher biogenic emissions in Houston from the subtropical region compared to New York City in the temperate region as monoterpene emission is temperature dependent.^64^ Assuming the anthropogenic emissions of monoterpenes remain similar between summer and winter, the results show that ∼75% of the summertime monoterpenes are attributed to biogenic sources in Houston and potentially in other subtropical cities.^65^

*para*-dichlorobenzene is mainly used as a pesticide. Concentrations of *para*-dichlorobenzene show a large spatial variation in both the winter and summer. The mean concentration was 3.8 ± 3.3 ppt in winter and 34.9 ± 22.6 ppt in summer. Summer concentrations of *para*-dichlorobenzene were significantly higher than those in the winter at the 5% significance level using a two-sample *t* test. The higher concentrations in the summer were likely due to the seasonal use of pesticides containing *para*-dichlorobenzene, and this pattern is comparable to other cities.^6^ However, the summer concentrations are about four times higher than in New York City, potentially due to the higher use of *para*-dichlorobenzene containing pesticides in subtropical areas. Additionally, higher concentrations were seen in areas with higher populations and decreased further south of the main metropolitan area. The range for summer and winter emissions is similar to indoor exposure estimates and is about half of the emission estimate for New York City.^6,66^ However, the summer concentrations of *para*-dichlorobenzene are about 4 times higher than those measured in New York City.

PCBTF is found in paints, caulking, solvent-based coatings, and cleaning products. The mean concentration in the winter was 7.9 ± 32.2 ppt, within a similar range of concentrations observed in other metropolitan areas, including New York City, Pittsburgh, Chicago, and Denver.^6,58^ The summertime concentration in Houston was 20.5 ± 35.2 ppt, with an average value 60% higher than those measured in New York City. Such a difference in concentrations is potentially due to heavier industrial activity in Houston.^6^ Considering the variation, the concentrations of PCBTF in Houston during the winter and summer were not statistically significantly different, likely due to the consistent use of products containing PCBTF throughout the year. Additionally, areas with elevated concentrations in both winter and summer are observed in the North-West as highlighted in Figure 1. Areas of elevated concentrations could indicate point sources, such as manufacturing facilities. Unlike monoterpene, D5-siloxane, and *para*-dichlorobenzene, areas of enhanced PCBTF concentrations seem to be driven by industrial use, such as construction or manufacturing, and not driven by population. Figure 2 demonstrates that PCBTF concentrations spike quickly and then decrease as the ROAM-V was moving across Houston, which indicates strong point sources, as such a spatial distribution profile was less commonly seen for the other measured compounds. The average emission rates were 13 mg person^–1^ day^–1^ and 59 mg person^–1^ day^–1^ for the winter and summer, respectively. Considering that emissions are calculated from the average daily concentration, the calculated emission of PCBTF may be significantly different depending on the proximity to point sources. For example, emissions in New York were four times higher in winter and 1.2 times higher in summer.^6^

Similar to PCBTF, Texanol is found in water-based coatings and typically occurs in industrialized areas. Here, the reported values of Texanol are calculated based on the base peak fragment, C_12_H_23_O_2_^+^, consistent with other studies.^6,58^ The mean concentration in winter was 72.4 ± 50.0 ppt, and in summer it was 200.1 ± 99.2 ppt. Summer concentrations are significantly higher possibly due to higher temperatures causing more evaporation or due to increased use in the summer.^58,67^ The concentrations of Texanol measured in this study are four to ten times higher than those previously reported.^6,58^ It is also possible that the higher concentrations and emissions for C_12_H_23_O_2_^+^ are due to other compounds with the same chemical formula or oxidation products, such as Rosamusk, a type of fragrance reported previously.^68^ It is worth noting that the concentrations of other VCP tracers reported in this study agree with values reported from previous measurements in different cities.^6^ As a result of the higher concentrations, the emissions for Texanol are also higher than those seen in New York City. The emissions for Texanol are 99 mg person^–1^ day^–1^ and 297 mg person^–1^ day^–1^ for winter and summer, respectively. High emissions of industrial compounds such as Texanol could be due to Houston having more industrial infrastructure and manufacturing compared with an urban center like Manhattan.

Considering that many VCPs exhibited higher concentrations in the Ship Channel area (Figure 1), further analysis was done to determine the influence of the Ship Channel on Houston Emissions. When winds originate from the east or southeast, emissions from the Ship Channel can be transported into the metropolitan area. While winds do not always come from the east or southeast, Ship Channel emissions have the potential to impact Houston under favorable wind conditions. To assess the impact of the Ship Channel on emission estimates, we calculated emissions separately for days when the wind direction favored transport from the Ship Channel to the metropolitan area and for days when it did not. Table S6 of the SI shows the comparison between total emissions and those including and excluding days favorable to Ship Channel influence. The comparison suggests that certain VCPs were enhanced on days influenced by Ship Channel emissions. Notably, emissions of PCBTF and *para*-dichlorobenzene were elevated when winds originated from the Ship Channel, indicating a potential source contribution.

### 3.4 Implications

This study provides the first temporally and spatially resolved VCP concentrations in Houston. The results can be used to understand VCP concentrations in urban areas within the subtropical United States. The two field campaigns in the winter and summer demonstrate the seasonal differences in VCP concentration as higher concentrations were observed for all compounds in the summer. This study also provides a thorough estimate of average VCP concentrations in the area and helps to identify point sources and areas that exhibit consistently elevated concentrations. The average and standard deviation obtained from the mobile measurements provide a more thorough evaluation of the GHA and are more representative than the use of only stationary measurements. By combining the average concentration and the loss pathways for OH· and O_3_, as well as the deposition process, this study obtained an estimation for VCP emissions that are representative of the GHA. Previous emission calculations typically use the ratios of VOCs with compounds common in emission inventories, such as CO, NO_*x*_, and benzene.^6,56^ Emissions are then calculated based on this ratio and the emission of a given species in the inventory. The two compounds must have some correlation to obtain the ratio between a given VOC and the inventory species. Therefore, this method is typically only applied to stationary measurements, as finding such correlations in mobile measurements can be difficult. Relying only on stationary measurements may be impacted by nearby point sources, especially for certain categories of VCPs, as demonstrated in this study. Additionally, the ratio method does not account for OH· or O_3_ reactivity of VOCs and assumes that the stationary site is close enough to the source that these reactions will play a minor role in the measured VOC concentrations.

In addition, the results reported here demonstrate that the concentrations and emissions of VCP in a subtropical city of Houston may be different when compared with previously reported VCP concentrations and emissions of cities in temperate climate zones. For example, the averaged summertime concentrations of monoterpene, *para*-dichlorobenzene, Texanol, and PCBTF in Houston were up to several times higher than New York City or other cities in the temperate region, while the averaged emissions of all key VCPs were higher than other cities reported. Such differences highlight the effects of population, industrial nature, and climate among different cities. Monoterpene and *para*-dichlorobenzene also demonstrate more biogenic emissions and seasonal use of pesticides during the summer compared to those in New York City, providing seasonal emission patterns that could be used to characterize emissions for other subtropical cities.

In summary, this research identifies the spatial and seasonal distributions of major tracer VCPs in Houston and provides a framework to determine VCP emissions from mobile data. Additionally, the box model used here can be applied to various locations and scales and may help determine the emission of other VOCs. This work provides the first study to show spatial and seasonal differences in both VCP concentration and emission in the subtropical climate zone, providing rationale to update emission inventories that may be underestimating the difference between summer and wintertime emissions in those areas.

## Supplementary Material



## Notes

**Section S1:** Field Deployment and Instrumentation; **Section S1.1:** Van Specifications, Figure S1: ROAM-V Layout; **Section S1.2:** Vocus-2R-CIMS Description; **Section S1.3**: Sampling Conditions, Table S1: Meteorology conditions for summer and winter deployments, Figure S2: Mobile measurement routes for winter and summer sampling periods; **Section S2:** VCP Measurements; **Section S2.1:** VCP Compounds and Calibrations, Table S2: Tracer VCPs with the formulas and fragments used in this work, Figure S3: Peak fittings for D5-siloxane, monoterpene, PCBTF, *para*-Dichlorobenzene, and Texanol, Figure S4: Laboratory calibrations for summer and winter, Figure S5: Field calibrations, Figure S6: Measured ion signal for each VCP compound for varying inlet tubing lengths; **Section S2.2:** VCP Concentration, Table S3: Mean and median concentrations for normalized and non-normalized concentrations, Figure S7: Houston population density, Figure S8: Hourly PBL height; **Section S3:** Emission Calculation, **Section S3.1:** Error propagation for emission; **Section S3.2:** Reaction Rates, Table S4: Reaction rates; **Section S3.3:** Community Multiscale Air Quality (CMAQ) and Weather Research and Forecast (WRF) Modeling, Figure S9: Location of air quality and meteorology stations for model performance evaluations, Figure S10: Measured and modeled January NO_2_ concentrations at four representative monitoring sites in Houston, Figure S11: Measured and modeled August NO_2_ concentrations at four representative monitoring sites in Houston, Figure S12: Measured and modeled January O_3_ concentrations at four representative monitoring sites in Houston, Figure S13: Measured and modeled August O_3_ concentrations at four representative monitoring sites in Houston, Figure S14: Measured and modeled January surface meteorological parameters at an urban site in Houston, Figure S15: Measured and modeled August surface meteorological parameters at an urban site in Houston, Table S5: Meteorology evaluation statistics for January and August 2023 for all available National Oceanic and Atmospheric Administration (NOAA) stations in the model domain. Figure S16: Predicted episode average O_3_ concentrations in the Houston area, Figure S17: Predicted episode average OH concentrations in the Houston area, Figure S18: Predicted episode average Planetary Boundary Layer (PBL) height in the Houston area; **Section S3.4:** Concentrations Reported by Mobile Measurement, Figure S19: Normalized VCP spatial distribution of VCPs, Figure S20: Non-normalized VCP time series for major VCPs, **Section S3.5:** VCP Emissions and Comparisons, Table S6: Emission rates for VCP compounds. Table S7: Concentration comparisons with previous work. Table S8: Emission rate comparison with previous work (PDF)

## ●

Civil and Environmental Engineering Department, University of Michigan, Ann Arbor, Michigan 48109, United States

## References

[1] Cohen A. J., Ross Anderson H., Ostro B., Pandey K. D., Krzyzanowski M., Künzli N., Gutschmidt K., Pope A., Romieu I., Samet J. M., Smith K. (2005). The Global Burden of Disease Due
to Outdoor Air Pollution. J. Toxicol. Environ.
Health, A.

[2] Landrigan P. J., Fuller R., Hu H., Caravanos J., Cropper M. L., Hanrahan D., Sandilya K., Chiles T. C., Kumar P., Suk W. A. (2018). Pollution and Global Health –
An Agenda for Prevention. Environ. Health Perspect..

[3] Li G., Wei W., Shao X., Nie L., Wang H., Yan X., Zhang R. (2018). A comprehensive classification
method for VOC emission sources to
tackle air pollution based on VOC species reactivity and emission
amounts. Journal of Environmental Sciences.

[4] Nault B. A., Jo D. S., McDonald B. C., Campuzano-Jost P., Day D. A., Hu W., Schroder J. C., Allan J., Blake D. R., Canagaratna M. R., Coe H., Coggon M. M., DeCarlo P. F., Diskin G. S., Dunmore R., Flocke F., Fried A., Gilman J. B., Gkatzelis G., Hamilton J. F., Hanisco T. F., Hayes P. L., Henze D. K., Hodzic A., Hopkins J., Hu M., Huey L. G., Jobson B. T., Kuster W. C., Lewis A., Li M., Liao J., Nawaz M. O., Pollack I. B., Peischl J., Rappenglück B., Reeves C. E., Richter D., Roberts J. M., Ryerson T. B., Shao M., Sommers J. M., Walega J., Warneke C., Weibring P., Wolfe G. M., Young D. E., Yuan B., Zhang Q., de Gouw J. A., Jimenez J. L. (2021). Secondary
organic aerosols from anthropogenic volatile organic compounds contribute
substantially to air pollution mortality. Atmos.
Chem. Phys..

[5] Nurmatov U. B., Tagiyeva N., Semple S., Devereux G., Sheikh A. (2015). Volatile organic
compounds and risk of asthma and allergy: a systematic review. Eur. Respir Rev..

[6] Gkatzelis G. I., Coggon M. M., McDonald B. C., Peischl J., Aikin K. C., Gilman J. B., Trainer M., Warneke C. (2021). Identifying Volatile
Chemical Product Tracer Compounds in U.S. Cities. Environ. Sci. Technol..

[7] Guenther A. B., Jiang X., Heald C. L., Sakulyanontvittaya T., Duhl T., Emmons L. K., Wang X. (2012). The Model
of Emissions
of Gases and Aerosols from Nature version 2.1 (MEGAN2.1): an extended
and updated framework for modeling biogenic emissions. Geosci. Model Dev..

[8] Jerrett M., Burnett R. T., Pope C. A., Ito K., Thurston G., Krewski D., Shi Y., Calle E., Thun M. (2009). Long-term ozone exposure and mortality. N Engl
J. Med..

[9] Jimenez J. L., Canagaratna M. R., Donahue N. M., Prevot A. S., Zhang Q., Kroll J. H., DeCarlo P. F., Allan J. D., Coe H., Ng N. L., Aiken A. C., Docherty K. S., Ulbrich I. M., Grieshop A. P., Robinson A. L., Duplissy J., Smith J. D., Wilson K. R., Lanz V. A., Hueglin C., Sun Y. L., Tian J., Laaksonen A., Raatikainen T., Rautiainen J., Vaattovaara P., Ehn M., Kulmala M., Tomlinson J. M., Collins D. R., Cubison M. J., Dunlea E. J., Huffman J. A., Onasch T. B., Alfarra M. R., Williams P. I., Bower K., Kondo Y., Schneider J., Drewnick F., Borrmann S., Weimer S., Demerjian K., Salcedo D., Cottrell L., Griffin R., Takami A., Miyoshi T., Hatakeyama S., Shimono A., Sun J. Y., Zhang Y. M., Dzepina K., Kimmel J. R., Sueper D., Jayne J. T., Herndon S. C., Trimborn A. M., Williams L. R., Wood E. C., Middlebrook A. M., Kolb C. E., Baltensperger U., Worsnop D. R. (2009). Evolution of organic aerosols in the atmosphere. Science.

[10] Yuan B., Shao M., de Gouw J., Parrish D. D., Lu S., Wang M., Zeng L., Zhang Q., Song Y., Zhang J., Hu M. (2012). Volatile organic
compounds (VOCs)
in urban air: How chemistry affects the interpretation of positive
matrix factorization (PMF) analysis. J. Geophys.
Res. (Atmos.).

[11] Brinke J. T., Selvin S., Hodgson A. T., Fisk W. J., Mendell M. J., Koshland C. P., Daisey J. M. (1998). Development of New Volatile Organic
Compound (VOC) Exposure Metrics and their Relationship to “Sick
Building Syndrome” Symptoms. Indoor Air.

[12] Dodson R. E., Nishioka M., Standley L. J., Perovich L. J., Brody J. G., Rudel R. A. (2012). Endocrine disruptors and asthma-associated chemicals
in consumer products. Environ. Health Perspect.

[13] Elberling J., Linneberg A., Dirksen A., Johansen J. D., Frølund L., Madsen F., Nielsen N. H., Mosbech H. (2005). Mucosal symptoms elicited
by fragrance products in a population-based sample in relation to
atopy and bronchial hyper-reactivity. Clin.
Exp. Allergy.

[14] Gabb H. A., Blake C. (2016). An Informatics Approach
to Evaluating Combined Chemical Exposures
from Consumer Products: A Case Study of Asthma-Associated Chemicals
and Potential Endocrine Disruptors. Environ.
Health Perspect.

[15] Trantallidi M., Dimitroulopoulou C., Wolkoff P., Kephalopoulos S., Carrer P. (2015). EPHECT III: Health
risk assessment of exposure to household
consumer products. Sci. Total Environ..

[16] Derwent R. G., Jenkin M. E., Utembe S. R., Shallcross D. E., Murrells T. P., Passant N. R. (2010). Secondary organic
aerosol formation
from a large number of reactive man-made organic compounds. Sci. Total Environ..

[17] Gentner D. R., Jathar S. H., Gordon T. D., Bahreini R., Day D. A., El Haddad I., Hayes P. L., Pieber S. M., Platt S. M., de Gouw J., Goldstein A. H., Harley R. A., Jimenez J. L., Prévôt A. S. H., Robinson A. L. (2017). Review of Urban
Secondary Organic Aerosol Formation from Gasoline and Diesel Motor
Vehicle Emissions. Environ. Sci. Technol..

[18] Gilman J. B., Lerner B. M., Kuster W. C., de Gouw J. A. (2013). Source signature
of volatile organic compounds from oil and natural gas operations
in northeastern Colorado. Environ. Sci. Technol..

[19] Kim S.-W., McKeen S., Frost G., Lee S.-H., Trainer M., Richter A., Angevine W., Atlas E., Bianco L., Boersma K., Brioude J., Burrows J., de Gouw J., Fried A., Gleason J., Hilboll A., Mellqvist J., Peischl J., Richter D., Williams E. (2011). Evaluations
of NOx and highly reactive VOC emission inventories in Texas and their
implications for ozone plume simulations during the Texas Air Quality
Study 2006. Atmospheric Chemistry and Physics.

[20] Ortega A. M., Hayes P. L., Peng Z., Palm B. B., Hu W., Day D. A., Li R., Cubison M. J., Brune W. H., Graus M., Warneke C., Gilman J. B., Kuster W. C., de Gouw J., Gutiérrez-Montes C., Jimenez J. L. (2016). Real-time
measurements of secondary organic aerosol formation and aging from
ambient air in an oxidation flow reactor in the Los Angeles area. Atmos. Chem. Phys..

[21] Ryerson T., Trainer M., Angevine W., Brock C. A., Dissly R., Fehsenfeld F., Frost G., Goldan P., Holloway J., Hübler G., Jakoubek R., Kuster W., Neuman J., Nicks D., Parrish D., Roberts J., Sueper D. (2003). Effect
of petrochemical industrial emissions of reactive alkenes
and NOx on tropospheric ozone formation in Houston, Texas. Journal Of Geophysical Research-Atmospheres.

[22] Seltzer K. M., Pennington E., Rao V., Murphy B. N., Strum M., Isaacs K. K., Pye H. O. T. (2021). Reactive
organic carbon emissions
from volatile chemical products. Atmos. Chem.
Phys..

[23] Dollard G., Dumitrean P., Telling S., Dixon J., Derwent R. (2007). Observed trends
in ambient concentrations of C2–C8 hydrocarbons in the United
Kingdom over the period from 1993 to 2004. Atmos.
Environ..

[24] Grant A., Yates E. L., Simmonds P. G., Derwent R. G., Manning A. J., Young D., Shallcross D. E., O’Doherty S. (2011). A five year
record of high-frequency in situ measurements of non-methane hydrocarbons
at Mace Head, Ireland. Atmos. Meas. Technol..

[25] McDonald B. C., Gentner D. R., Goldstein A. H., Harley R. A. (2013). Long-term trends
in motor vehicle emissions in u.s. urban areas. Environ. Sci. Technol..

[26] Warneke C., de Gouw J. A., Holloway J. S., Peischl J., Ryerson T. B., Atlas E., Blake D., Trainer M., Parrish D. D. (2012). Multiyear
trends in volatile organic compounds in Los Angeles, California: Five
decades of decreasing emissions. Journal of
Geophysical Research: Atmospheres.

[27] McDonald B. C., de Gouw J. A., Gilman J. B., Jathar S. H., Akherati A., Cappa C. D., Jimenez J. L., Lee-Taylor J., Hayes P. L., McKeen S. A., Cui Y. Y., Kim S. W., Gentner D. R., Isaacman-VanWertz G., Goldstein A. H., Harley R. A., Frost G. J., Roberts J. M., Ryerson T. B., Trainer M. (2018). Volatile chemical products emerging
as largest petrochemical
source of urban organic emissions. Science.

[28] Coggon M. M., Gkatzelis G. I., McDonald B. C., Gilman J. B., Schwantes R. H., Abuhassan N., Aikin K. C., Arend M. F., Berkoff T. A., Brown S. S., Campos T. L., Dickerson R. R., Gronoff G., Hurley J. F., Isaacman-VanWertz G., Koss A. R., Li M., McKeen S. A., Moshary F., Peischl J., Pospisilova V., Ren X., Wilson A., Wu Y., Trainer M., Warneke C. (2021). Volatile chemical product emissions
enhance ozone and modulate urban chemistry. Proc. Natl. Acad. Sci. U. S. A..

[29] Pennington E. A., Seltzer K. M., Murphy B. N., Qin M., Seinfeld J. H., Pye H. O. T. (2021). Modeling secondary organic aerosol
formation from volatile
chemical products. Atmos. Chem. Phys..

[30] Humes M. B., Wang M., Kim S., Machesky J. E., Gentner D. R., Robinson A. L., Donahue N. M., Presto A. A. (2022). Limited Secondary
Organic Aerosol Production from Acyclic Oxygenated Volatile Chemical
Products. Environ. Sci. Technol..

[31] Nematollahi N., Kolev S., Steinemann A. (2019). Volatile chemical emissions from
134 common consumer products. Air Quality, Atmosphere
& Health.

[32] Qin M., Murphy B. N., Isaacs K. K., McDonald B. C., Lu Q., McKeen S. A., Koval L., Robinson A. L., Efstathiou C., Allen C., Pye H. O. T. (2021). Criteria pollutant impacts of volatile
chemical products informed by near-field modelling. Nature Sustainability.

[33] Wolkoff P. (1998). Impact of
air velocity, temperature, humidity, and air on long-term voc emissions
from building products. Atmos. Environ..

[34] Cai Z., Xie Q., Yang L., Yuan B., Wu G., Zhu Z., Wu L., Chang M., Wang X. (2023). A novel method for spatial allocation
of volatile chemical products emissions: A case study of the Pearl
River Delta. Atmos. Environ..

[35] Jung Y., Kim Y., Seol H.-S., Lee J.-H., Kwon J.-H. (2021). Spatial Uncertainty
in Modeling Inhalation Exposure to Volatile Organic Compounds in Response
to the Application of Consumer Spray Products. International Journal of Environmental Research and Public Health.

[36] Zhu Y., Guo S., Liang W. (2024). A literature
review investigating the impact of temperature
and humidity on volatile organic compound emissions from building
materials. Building and Environment.

[37] Liu C.-C., Chen W.-H., Yuan C.-S., Lin C. (2014). Multivariate analysis
of effects of diurnal temperature and seasonal humidity variations
by tropical savanna climate on the emissions of anthropogenic volatile
organic compounds. Science of The Total Environment.

[38] Baudic A., Gros V., Sauvage S., Locoge N., Sanchez O., Sarda-Estève R., Kalogridis C., Petit J. E., Bonnaire N., Baisnée D., Favez O., Albinet A., Sciare J., Bonsang B. (2016). Seasonal variability and source apportionment of volatile
organic compounds (VOCs) in the Paris megacity (France). Atmos. Chem. Phys..

[39] Lewis A. C., Hopkins J. R., Carslaw D. C., Hamilton J. F., Nelson B. S., Stewart G., Dernie J., Passant N., Murrells T. (2020). An increasing
role for solvent emissions and implications for future measurements
of volatile organic compounds. Philos. Trans
A Math Phys. Eng. Sci..

[40] 200 Largest Cities in the United States by Population 2024. 2024. https://worldpopulationreview.com/us-cities (accessed 12 Dec 2023).

[41] Krechmer J., Lopez-Hilfiker F., Koss A., Hutterli M., Stoermer C., Deming B., Kimmel J., Warneke C., Holzinger R., Jayne J., Worsnop D., Fuhrer K., Gonin M., de Gouw J. (2018). Evaluation of a New Reagent-Ion Source and Focusing
Ion–Molecule Reactor for Use in Proton-Transfer-Reaction Mass
Spectrometry. Anal. Chem..

[42] Blake R. S., Monks P. S., Ellis A. M. (2009). Proton-Transfer
Reaction Mass Spectrometry. Chem. Rev..

[43] de
Gouw J., Warneke C. (2007). Measurements of volatile organic compounds in the earth’s
atmosphere using proton-transfer-reaction mass spectrometry. Mass Spectrom Rev..

[44] Lindinger W., Hansel A., Jordan A. (1998). On-line monitoring
of volatile organic
compounds at pptv levels by means of proton-transfer-reaction mass
spectrometry (PTR-MS) medical applications, food control and environmental
research. International Journal of Mass Spectrometry
and Ion Processes.

[45] Krechmer J., Lopez-Hilfiker F., Koss A., Hutterli M., Stoermer C., Deming B., Kimmel J., Warneke C., Holzinger R., Jayne J., Worsnop D., Fuhrer K., Gonin M., de Gouw J. (2018). Evaluation of a New Reagent-Ion Source
and Focusing
Ion–Molecule Reactor for Use in Proton-Transfer-Reaction Mass
Spectrometry. Anal. Chem..

[46] Stark H., Yatavelli R. L. N., Thompson S. L., Kimmel J. R., Cubison M. J., Chhabra P. S., Canagaratna M. R., Jayne J. T., Worsnop D. R., Jimenez J. L. (2015). Methods
to extract molecular and bulk chemical information
from series of complex mass spectra with limited mass resolution. Int. J. Mass Spectrom..

[47] Brunet C. E., Marek R. F., Stanier C. O., Hornbuckle K. C. (2024). Concentrations
of Volatile Methyl Siloxanes in New York City Reflect Emissions from
Personal Care and Industrial Use. Environ. Sci.
Technol..

[48] Tang X., Misztal P. K., Nazaroff W. W., Goldstein A. H. (2015). Siloxanes
Are the Most Abundant Volatile Organic Compound Emitted from Engineering
Students in a Classroom. Environmental Science
& Technology Letters.

[49] Hantson S., Knorr W., Schurgers G., Pugh T. A. M., Arneth A. (2017). Global isoprene
and monoterpene emissions under changing climate, vegetation, CO2
and land use. Atmos. Environ..

[50] Li F., Huang D. D., Tian L., Yuan B., Tan W., Zhu L., Ye P., Worsnop D., Hoi K. I., Mok K. M., Li Y. J. (2024). Response
of protonated, adduct, and fragmented ions in Vocus proton-transfer-reaction
time-of-flight mass spectrometer (PTR-ToF-MS). Atmos. Meas. Technol..

[51] Houston, TX Weather History. https://www.wunderground.com/history/daily/us/tx/houston/KHOU/date/2023-8-10 (accessed 12 Aug 2024).

[52] Agency, U. S. E. PA . CMAQ, Version 5.3.2. 2020. 10.5281/zenodo.4081737 (accessed 15 Sep 2024).

[53] Wang P., Chen Y., Hu J., Zhang H., Ying Q. (2019). Source apportionment
of summertime ozone in China using a source-oriented chemical transport
model. Atmos. Environ..

[54] Wang P., Chen Y., Hu J., Zhang H., Ying Q. (2019). Attribution
of Tropospheric Ozone to NO (x) and VOC Emissions: Considering Ozone
Formation in the Transition Regime. Environ.
Sci. Technol..

[55] Partnership G H Houston metro area had the second largest numeric population growth in the country between ’22 and ’23. 2024. https://www.houston.org/houston-data/population-growth (accessed 12 Dec 2023).

[56] Coggon M.
M., McDonald B. C., Vlasenko A., Veres P. R., Bernard F., Koss A. R., Yuan B., Gilman J. B., Peischl J., Aikin K. C., DuRant J., Warneke C., Li S.-M., de Gouw J. A. (2018). Diurnal
Variability and Emission Pattern of Decamethylcyclopentasiloxane
(D5) from the Application of Personal Care Products in Two North American
Cities. Environ. Sci. Technol..

[57] Hegar, G. Port of Entry: Houston-Port of Houston Economic Impact, 2015. 2015. https://comptroller.texas.gov/economy/economic-data/ports/2016/houston.php#:~:text=The%20ship%20channel%20officially%20opened,230%20million%20tons%20of%20cargo. (accessed 15 Oct 2023).

[58] Stockwell C. E., Coggon M. M., Gkatzelis G. I., Ortega J., McDonald B. C., Peischl J., Aikin K., Gilman J. B., Trainer M., Warneke C. (2021). Volatile organic
compound emissions from solvent- and
water-borne coatings – compositional differences and tracer
compound identifications. Atmos. Chem. Phys..

[59] Seltzer K. M., Murphy B. N., Pennington E. A., Allen C., Talgo K., Pye H. O. T. (2022). Volatile Chemical Product Enhancements to Criteria
Pollutants in the United States. Environ. Sci.
Technol..

[60] Ahrens L., Harner T., Shoeib M. (2014). Temporal variations of cyclic and
linear volatile methylsiloxanes in the atmosphere using passive samplers
and high-volume air samplers. Environ. Sci.
Technol..

[61] Buser A. M., Kierkegaard A., Bogdal C., MacLeod M., Scheringer M., Hungerbühler K. (2013). Concentrations in Ambient Air and Emissions of Cyclic
Volatile Methylsiloxanes in Zurich, Switzerland. Environ. Sci. Technol..

[62] Buser A. M., Bogdal C., MacLeod M., Scheringer M. (2014). Emissions
of decamethylcyclopentasiloxane from Chicago. Chemosphere.

[63] Administration, N. O. A. A . National Weather Service, 2022. https://www.weather.gov/climateservices/nowdatafaq (accessed 22 May 2024).

[64] Bourtsoukidis E., Pozzer A., Williams J., Makowski D., Peñuelas J., Matthaios V. N., Lazoglou G., Yañez-Serrano A. M., Lelieveld J., Ciais P., Vrekoussis M., Daskalakis N., Sciare J. (2024). High temperature sensitivity of monoterpene
emissions from global vegetation. Communications
Earth & Environment.

[65] Peng Y., Mouat A. P., Hu Y., Li M., McDonald B. C., Kaiser J. (2022). Source appointment of volatile organic
compounds and
evaluation of anthropogenic monoterpene emission estimates in Atlanta,
Georgia. Atmos. Environ..

[66] Nazaroff W., Weschler Charles J., Little John C., Hubal Elaine A. C. (2012). Intake
to Production Ratio: A Measure of Exposure Intimacy for Manufactured
Chemicals. Environ. Health Perspect..

[67] Ho K. F., Lee S. C., Guo H., Tsai W. Y. (2004). Seasonal and diurnal
variations of volatile organic compounds (VOCs) in the atmosphere
of Hong Kong. Science of The Total Environment.

[68] Chemist, A. ChemSpider SyntheticPages. 2024. http://cssp.chemspider.com/Search (accessed 22 May 2024).

