# Supplementary material for: Quantifying the Spatial and Temporal Distributions of Volatile Chemical Products (VCPs) in the Greater Houston Area
Source: Environ Sci Technol. 2025 Jun 26;59(27):13881–91. doi: 10.1021/acs.est.4c13855 (PMC12269074; doi:10.1021/acs.est.4c13855)
Supplement: Supplementary file 1 [file es4c13855_si_001.pdf]

Supporting Information

Quantifying the Spatial and Temporal Distributions of Volatile Chemical Products (VCPs) in the Greater Houston Area

Alana J. Dodero,[1] Sining Niu,[1] Heewon Yim,[2] Kyle P. McCary,[1] Sahir Gagan,[1] Yeaseul Kim[1,†], Timothy B. Onasch,[3] James H. Flynn,[4] Raghu Betha,[5] Karsten Baumann,[6, 7] Sarah D. Brooks,[1] Qi Ying,[2] Yue Zhang[1*]

[1]*Department of Atmospheric Sciences, Texas A&M University, College Station, TX, 77843, USA*

[2]*Department of Civil and Environmental Engineering, Texas A&M University, College Station, TX, 77843, USA*

[3]*Aerodyne Research, Billerica, MA, 01821, USA*

[4]*Institute for Climate and Atmospheric Science, University of Houston, Houston, TX, 77204, USA*

[5]*Department of Civil, Environmental, &; Construction Engineering, Texas Tech University, Lubbock, TX, 79409, USA*

[6]*Picarro, Inc., Santa Clara, CA, 95054, USA*

[7]*Department of Environmental Sciences and Engineering, University of North Carolina, Chapel Hill, NC, 27599, USA*

[†]*Now at Civil and Environmental Engineering Department, University of Michigan, Ann Arbor, MI, United States 48109*

June 2025

Environmental Science & Technology

[*]*Corresponding author:* Yue Zhang, yuezhang@tamu.edu

No. of pages: 29

No. of figures: 20

No. of tables: 8

S1. Field Deployment and Instrumentation

S1.1 Van Specifications

The Texas A&M ROAM-V is equipped with multiple gas-phase and aerosol-phase instruments. All instruments were connected to Wi-Fi during mobile measurements and were continuously monitored from the cabin. The TAMU ROAM-V is a 2018 Ford Transit HD-350 with four lithium-ion 48 V batteries and two air conditioners. The inlet of the van sits on top at a height of 3 meters and samples air at a flow rate of 5 liters per minute (LPM). Overall schematic view of the mobile lab is shown in Figure S1.

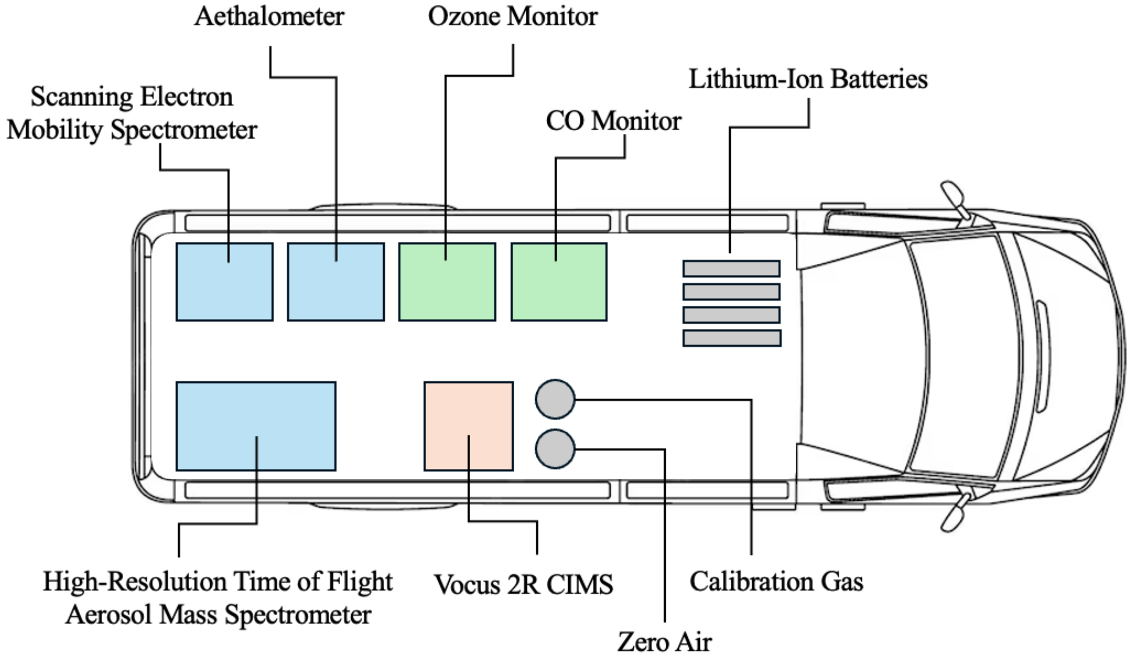

Figure S1. ROAM-V Layout. Gas phase monitors are shown in green, aerosol phase monitors in blue, and the Vocus 2R CIMS in red.

S1.2 Vocus 2R CIMS Description

The working principles of the Vocus 2R CIMS can be found in detail in Krechmer et al.[1] The Vocus 2R CIMS ran in H_3O^+ mode in this study. In short, the Vocus 2R CIMS uses a

quadrupole RF field in the reactor to collimate ions onto the central axis and improve the detection limit while maintaining similar collision conditions for conventional drift tubes.[1] The reagent-ion source consists of two conical surfaces between which a plasma is produced. The corresponding vapor enters the focusing ion-molecule reactor (FIMR). The discharged current is regulated to be 1.5 – 2.0 mA. The sampling air enters the FIMR with a pressure of 1 mbar, which is controlled by a valve between the reactor and a mechanical pump. The FIMR consists of a 10 cm glass tube and a homogeneous electric field will be generated. At the end of the FIMR, the adduct ions are sampled into a time-of-flight mass spectrometer for detection. The ions from FIMR first enter a big, segmented quadrupole (BSQ) region to focus the ion beam, and then enter the primary beam (PB) region, and finally the ToF chamber. The BSQ acts as a high-pass band filter that ions smaller than the specific mass-to-charge ratio (m/z) determined by the voltage setting (~35 Th)[1] will be detected at a much lower efficiency. Such a setting could help prevent the degradation of the microchannel plate detector due to the very high reagent ion intensities.

S1.3 Sampling Conditions

Meteorological data was obtained from the George Bush Intercontinental Airport (29.99° N, 95.34° W) and William P. Hobby Airport (29.38° N, -95.16° W). The ambient temperature, humidity, wind speed, and wind direction varied between mobile collection days and seasons. In particular, the wind direction varied significantly between days. Precipitation patterns were consistent with little to no precipitation.

Table S1. Measurement dates and times with average meteorology conditions for summer and winter deployments.

	Date	Measurement Time	Temperature (°C)	Humidity (%)	Wind Speed (ms^{-1})	Wind Direction
**Winter 2023**	17-01-2023	10:45-17:27	24.9 ± 1.1	64.9 ± 6.3	4.3 ± 1.3	SW
	18-01-2023	9:50-16:27	23.1 ± 2.1	71 ± 12.3	7 ± 2.7	Variable
	19-01-2023	11:42-19:03	23.5 ± 1.3	22.1 ± 2.2	4.8 ± 1.6	NNE
	22-01-2023	9:27-14:39	15.0 ± 2.0	38.1 ± 8.7	6.3 ± 0.5	NW
	23-01-2023	10:42-19:15	13.1 ± 1.3	42.8 ± 11.5	5.2 ± 1.2	E
	24-01-2023	10:20-19:05	16.2 ± 3.9	88.4 ± 3.9	9.2 ± 2.6	Variable
	January Average		15.9 ± 5.1	60.5 ± 9.2	4.5 ± 1.6	
**Summer 2023**	10-08-2023	6:07-11:10	32.1 ± 2.9	65.2 ± 15.5	6.4 ± 2.1	SSW
	16-08-2023	6:32-11:50	31.9 ± 2.4	38.7 ± 5.9	4.2 ± 1.5	E
	18-08-2023	11:22-15:11	37.1 ± 1.2	37.3 ± 3.9	3.2 ± 1.7	SE
	19-08-2023	6:58-12:29	31.9 ± 2.9	63.2 ± 18.1	2.4 ± 1.8	W
	26-08-2023	7:35-11:23	32.6 ± 2.4	60.8 ± 13.3	1.0 ± 0.7	Variable
	August Average		32.0 ± 0.7	61.2 ± 8.9	4.1 ± 1.0	

Two routes were completed for both Summer and Winter field campaigns. The round route, shown on the left panel of Figure S2, went through the downtown and metro areas of Houston. The North-South route went through areas of high population along with more rural areas, particularly to the South.

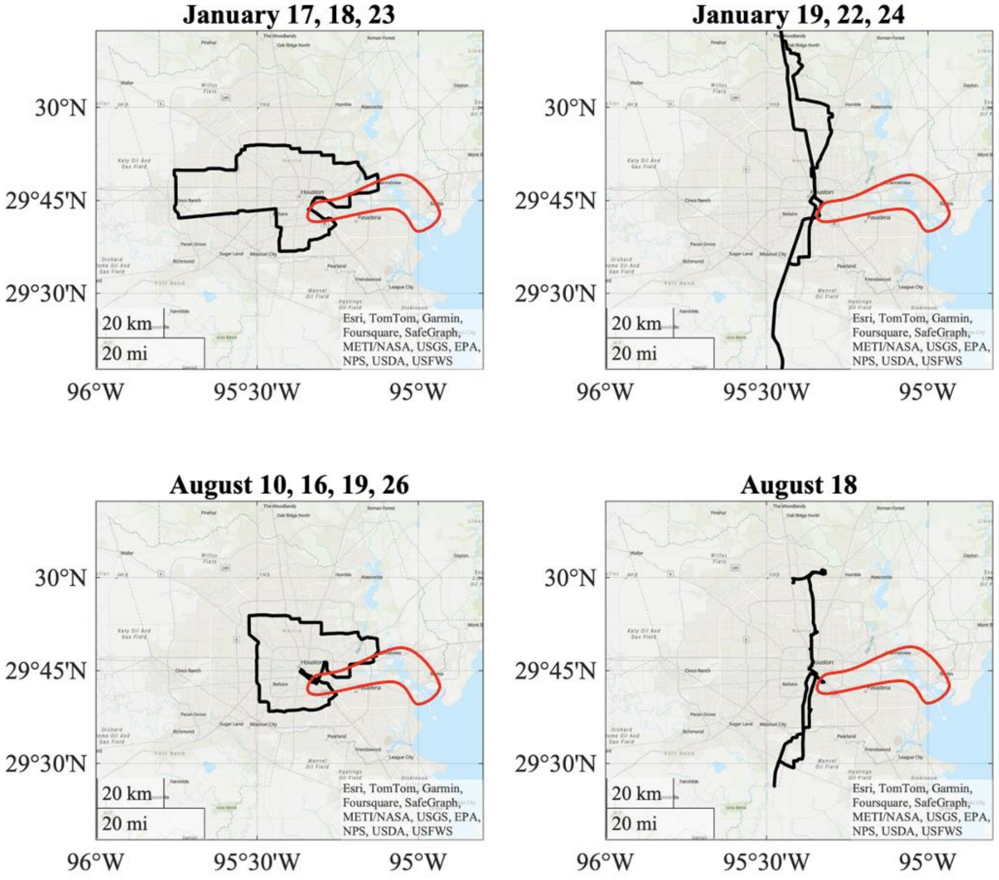

Figure S2. Mobile measurement routes for winter and summer sampling periods. The Ship Channel region is highlighted in red.

S2. VCP Measurements

S2.1 VCP Compounds and Calibrations

Most tracer compounds follow the reaction $H_3O^+ + R \rightarrow RH^+ + H_2O$ and were identified by the parent ion RH^+. However, fragmentation occurs with the Vocus 2R CIMS for both PCBTF and Texanol. For these compounds, the sensitivity to the fragment (base peak) was higher than that of the parent ion, therefore, the fragment peak was used, as shown in Table S2. Peak fitting results are shown in Figure S3. α-pinene was used to determine the concentrations for all monoterpenes, which may introduce uncertainties in the overall monoterpene estimates. α-pinene was used to

calibrate for monoterpenes due to its substantial contribution to total monoterpenes in ambient air. While monoterpenes in New York City were found to be dominated by limonene, the large biogenic sources in Houston were expected to contribute significantly to overall monoterpenes. Similarly, as the sensitivities of α-pinene and limonene are similar, their use in calibration would not significantly affect monoterpene concentration and emission estimates. Laboratory and field calibration results are shown in Figures S4-S5.

Table S2. Tracer VCPs molecular formulas and measured parent ions used in this work.

	**D5-siloxane**	**Monoterpene**	**PCBTF**	***p*-Dichlorobenzene**	**Texanol**
**Molecular Formula**	$C_{10}H_{30}O_5Si_5$	$C_{10}H_{16}$	$C_7H_4ClF_3$	$C_6H_4Cl_2$	$C_{12}H_{24}O_3$
**Measured Parent Ion Formula**	$C_{10}H_{30}O_5Si_5H\cdot^+$	$C_{10}H_{16}H\cdot^+$	$C_7H_3ClF_2H\cdot^+$	$C_6H_4Cl_2H\cdot^+$	$C_{12}H_{22}O_2H\cdot^+$
**Measured m/z**	371.101	137.133	160.997	146.976	199.169

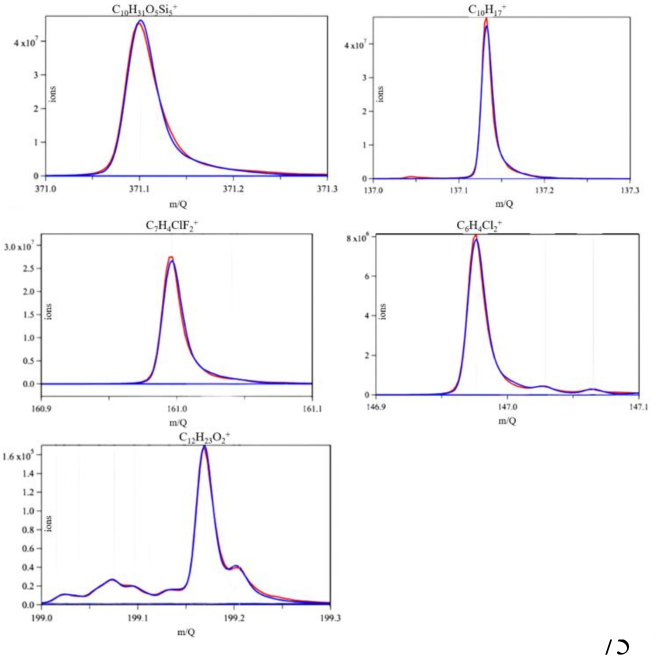

Figure S3. Peak fitting for (a) D5-siloxane, (b) monoterpene, (c) PCBTF, (d) *para*-Dichlorobenzene, and (e) Texanol using Igor Pro version 8, Tofware Analysis program version 3.2.3.

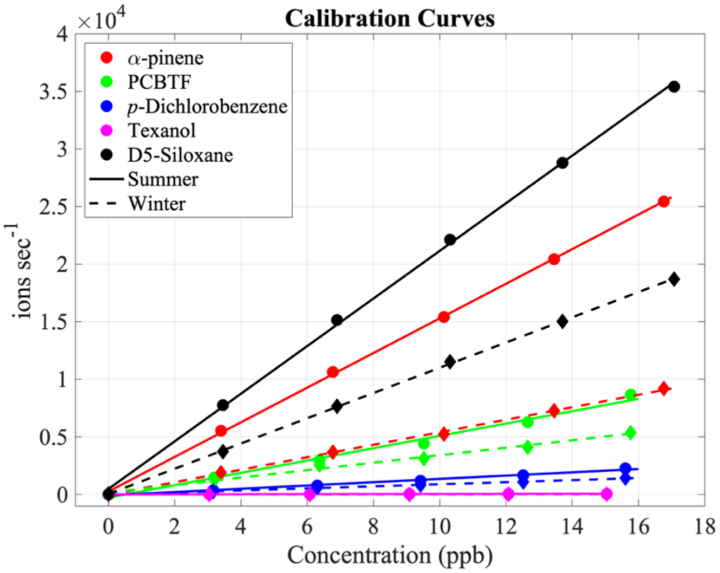

Figure S4. Laboratory calibrations for summer (solid line) and winter (dashed line).

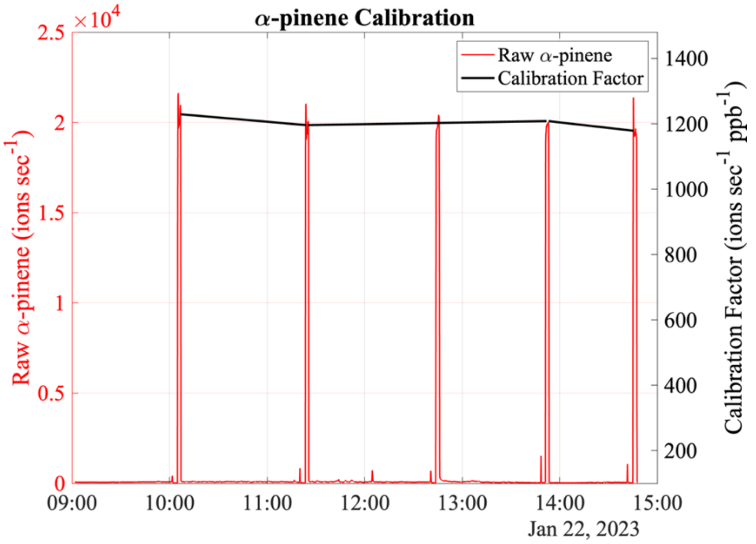

Figure S5. Field calibrations conducted (α-pinene as an example) is shown in red on the left axis. The calibration factor for α-pinene derived from the calibration is shown in black on the right axis.

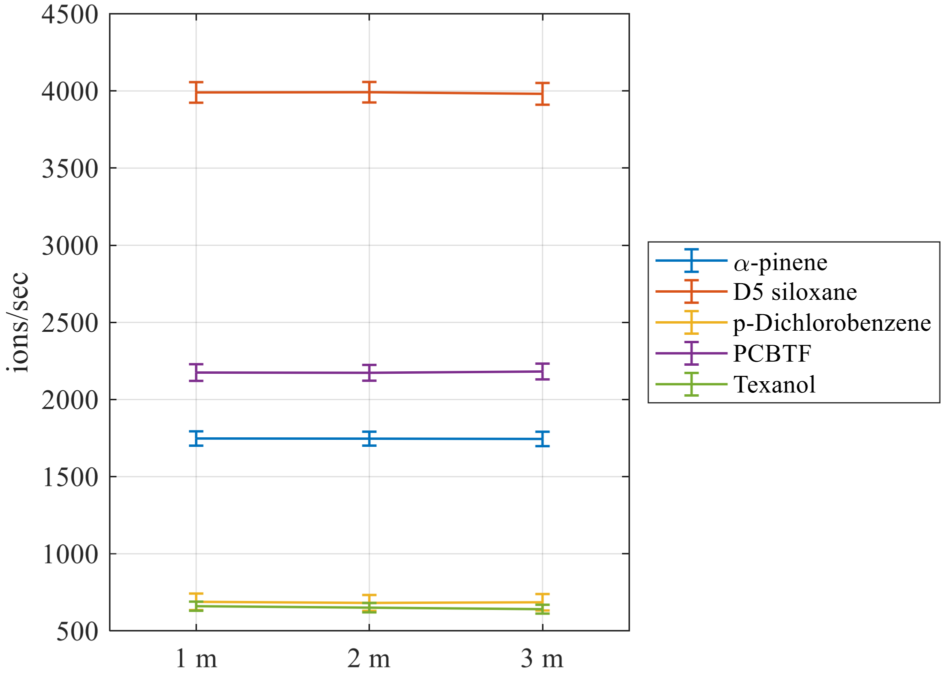

Figure S6. Measured ion signal for each VCP compound for varying inlet tubing lengths.

S2.2 VCP Concentration

Population density of Houston is shown in Figure S6. Given that the drives were completed throughout the day, the PBL height could lead to enhancements in concentrations in the morning and evening, as shown in Figure S7. Therefore, ambient VCP concentrations were normalized to hourly PBL height, as shown in Table S3 and Figure S8.

Table S3. Mean and median concentrations (ppt) for non-normalized concentrations (top) and normalized concentrations (bottom).

**Normalized Concentration**					
	**Monoterpene**	**D5-siloxane**	**PCBTF**	***p-Dichlorobenzene***	**Texanol**
**Winter mean**	74.9 ± 59.6	8.5 ± 5.2	7.9 ± 32.2	3.8 ± 3.3	72.4 ± 50.0
**Winter median**	56.9	7.4	2.9	3.1	56.4
**Summer mean**	249.6 ± 219.0	10.1 ± 11.3	20.5 ± 35.2	34.9 ± 22.6	200.1 ± 99.2
**Summer median**	194.1	5.9	12.7	31.1	189.9
**Non-normalized Concentration**					
**Winter mean**	77.9 ± 60.5	8.7 ± 5.1	7.9 ± 32.4	4.2 ± 4.6	71.9 ± 40.9
**Winter median**	56.7	8.3	3.3	3.1	64.2
**Summer mean**	297.1 ± 318.3	11.1 ± 11.8	20.9 ± 32.2	42.0 ± 41.9	210.1 ± 102.7
**Summer median**	220.2	6.4	12.1	32.1	191.8

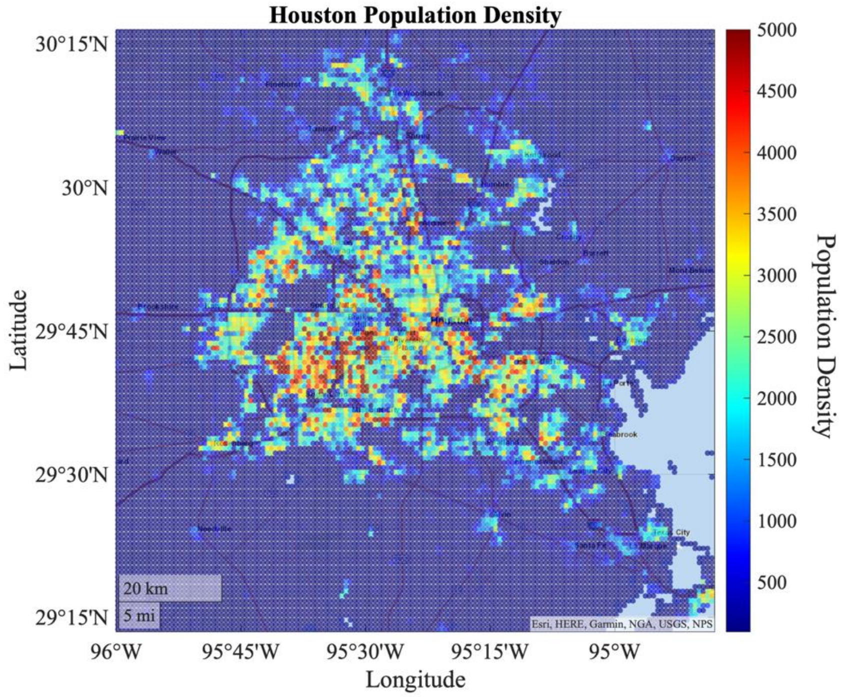

Figure S7. Houston population density obtained from WorldPop. Such data was used to calculate per capita emission values.

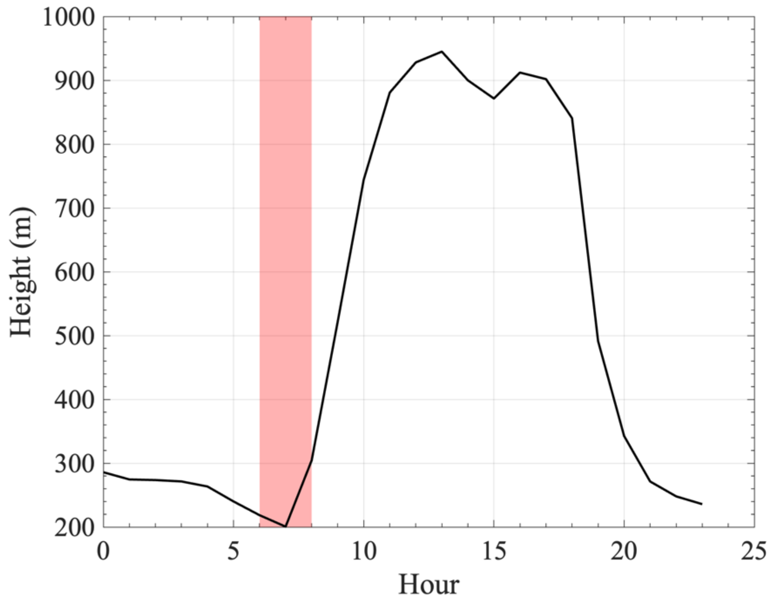

Figure S8. Hourly averaged summer PBL height. The hours when the mobile van was close to the Ship Channel are highlighted in red and show a lower PBL height in early morning.

S3. Emission Calculation

S3.1 Error propagation for emission

The emission equation is calculated as Eq. S1:

$$E = k_{OH}[OH]\left[C * \frac{H}{H_{avg}}\right]H + k_{O_3}[O_3][C]H + v_d[C]$$

(S1)

The error for emission is calculated using error propagation where the standard deviation is determined from the 24-hour time series for each term.

The error is calculated using Eqs. S2-S3:

$$err_1 = \left(\frac{\sigma_{k_{OH}}^2}{k_{OH}^2} + \frac{\sigma_{OH}^2}{OH^2} + \frac{\sigma_C^2}{C^2} + \frac{\sigma_H^2}{H^2}\right)(k_{OH}[OH][C]H)^2 \quad \text{(S2a)}$$

$$err_2 = \left(\frac{\sigma_{k_{O_3}}^2}{k_{O_3}^2} + \frac{\sigma_{O_3}^2}{O_3^2} + \frac{\sigma_C^2}{C^2} + \frac{\sigma_H^2}{H^2}\right)\left(k_{O_3}[O_3][C]H\right)^2 \quad \text{(S2b)}$$

$$err_3 = \left(\frac{\sigma_{v_d}^2}{v_d^2} + \frac{\sigma_C^2}{C^2}\right)(v_d[C])^2 \quad \text{(S2c)}$$

$$E_{err} = \sqrt{err_1 + err_2 + err_3} \quad \text{(S3)}$$

S3.2 Reaction Rates

Reaction rates for each VCP with OH· and O_3 were obtained from various lab studies, as shown in Table S4 with references included. The standard deviation for each reaction rate is either the published value or the square root of the reaction rate multiplied by 20%.

Table S4. Reaction rates used in the box model to calculate the emission values, with references included.

	D5-siloxane	Monoterpene	PCBTF	*p*-Dichlorobenzene	Texanol
k_{OH}	$(2.1 \pm 0.1) \times 10^{-12}$	$(6.01 \pm 0.82) \times 10^{-11}$	$(1.6 \pm 0.03) \times 10^{-13}$	$(4.3 \pm 0.9) \times 10^{-13}$	1.62×10^{-11}
k_{O3}	n/a	$(1.4 \pm 0.4) \times 10^{-15}$	5×10^{-23}	1.47×10^{-23}	n/a
Reference	Alton et al. (2020)[2]	Montenegro et al. (2012)[3], Tillmann et al. (2009)[4]	Chattopadhyay et al. (2022),[5] Atkinson et al. (1985)[6]	Arnts et al. (1988),[7] Wen et al. (2023)[8]	Cater et al. (2005)[9]

S3.3 Community Multiscale Air Quality (CMAQ) and Weather Research and Forecast (WRF) Modeling

The Community Multiscale Air Quality (CMAQ) model version 5.0.1 was used in this study.[10-12] The gas phase chemical mechanism is a detailed version of the SAPRC-11 mechanism (SAPRC-11D).[13, 14] Anthropogenic emissions were generated from the 2017 National Emission Inventory (NEI) using the input files from the Emission Modeling Platform provided by the US EPA[15] and processed using the Sparse Matrix Operator Kernel Emissions (SMOKE) from US EPA. Biogenic emissions were generated by the Model for Emissions of Gaseous and Aerosols from Nature (MEGAN) v 2.1.0.[16]

The Weather Research and Forecast (WRF) model version 4.5.1 was used to generate the meteorological inputs for the CMAQ model. The initial and boundary conditions for the WRF model were generated from the 1°x1^{o} National Centers for Environmental Prediction Final (NCEP FNL) reanalysis. The major physics options follow those used in Zhang et al.,[17] including the Yonsei University PBL scheme, RRTM shortwave radiation scheme, the Goddard shortwave

radiation scheme, and the MM5 land surface model. The single-layer urban surface model was used for the 4 and 1.33-km resolution domains.

Model performance evaluations along with model outputs for O_3, OH, and PBL are shown below. As shown in the time series plots of predicted and observed NO_2 and O_3 concentrations in Figures S11-S14, the WRF-CMAQ model can capture the diurnal variations of NO_2 and O_3 during the campaign periods, which provides confidence in the modelled OH radical concentrations. The overall model performance statistics of hourly O_3 in August are normalized mean bias (NMB) = -0.21, and normalized mean error (NME) = 0.22. Wintertime O_3 is slightly overpredicted on a few days. However, since the loss of the compounds via O_3 reactions is generally slow, it should not lead to significant biases in the subsequent analyses. The overall WRF model performance is shown in Table S4. In general, the WRF model performance is similar to previous studies.[17, 18] However, as shown in Figure S15 and Figure S16, wind speed in the urban area was slightly underestimated.

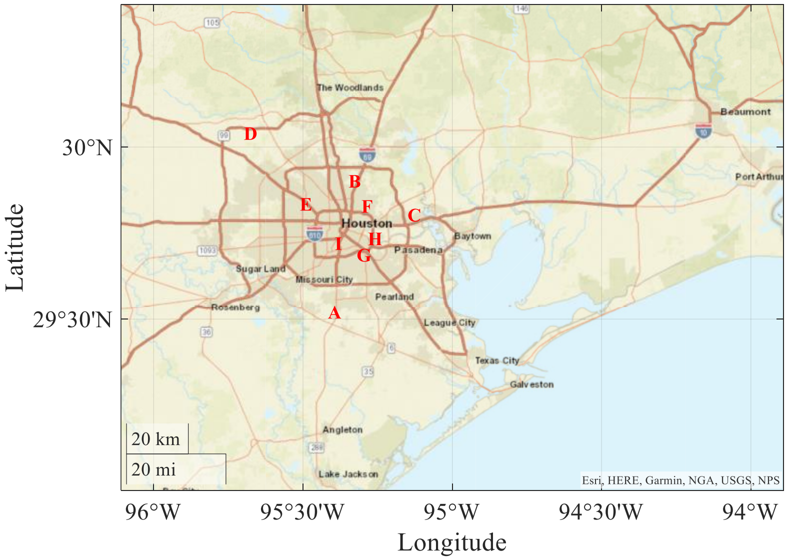

Figure S9. Location of the air quality and meteorology stations mentioned in the model performance evaluations (Figures S11-S14).

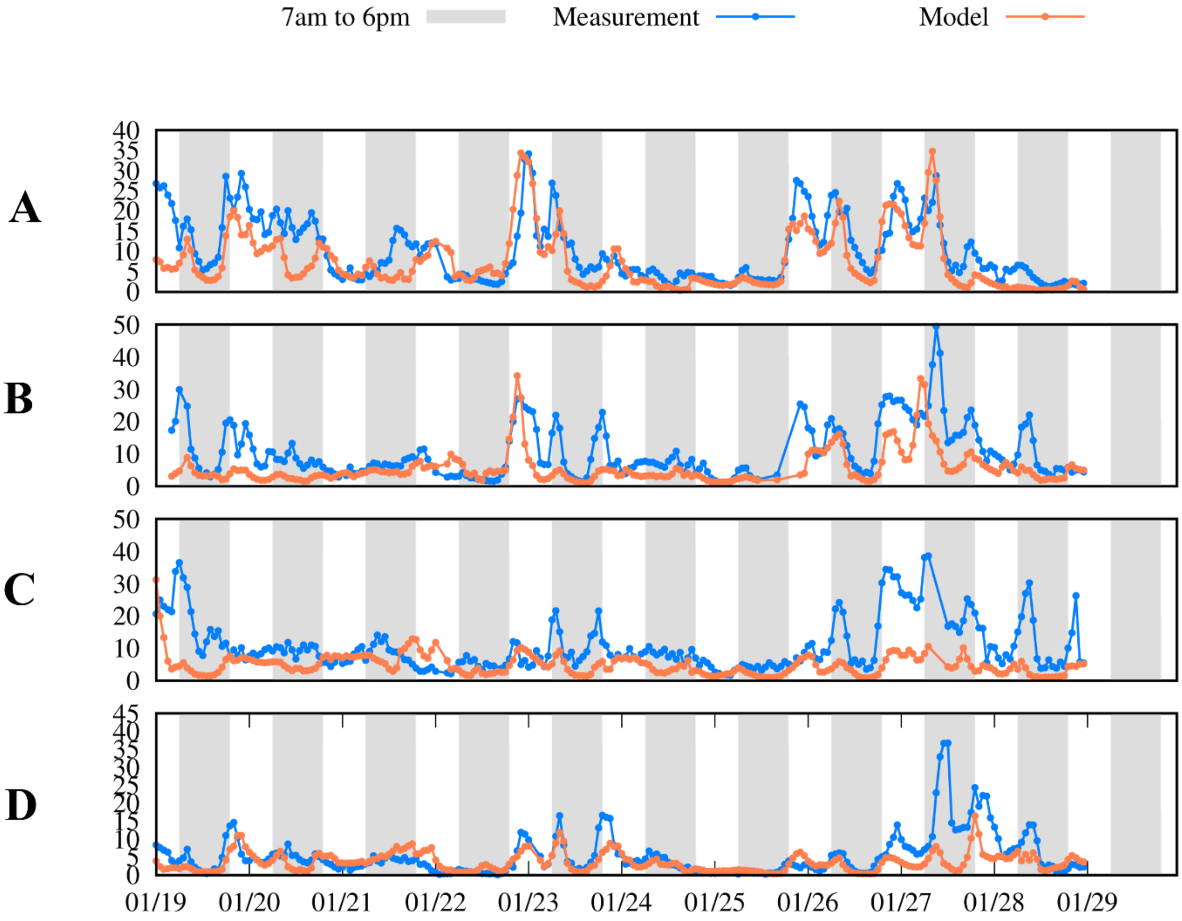

Figure S10. Measured and modelled January NO_2 concentrations at four representative monitoring sites in Houston.

158 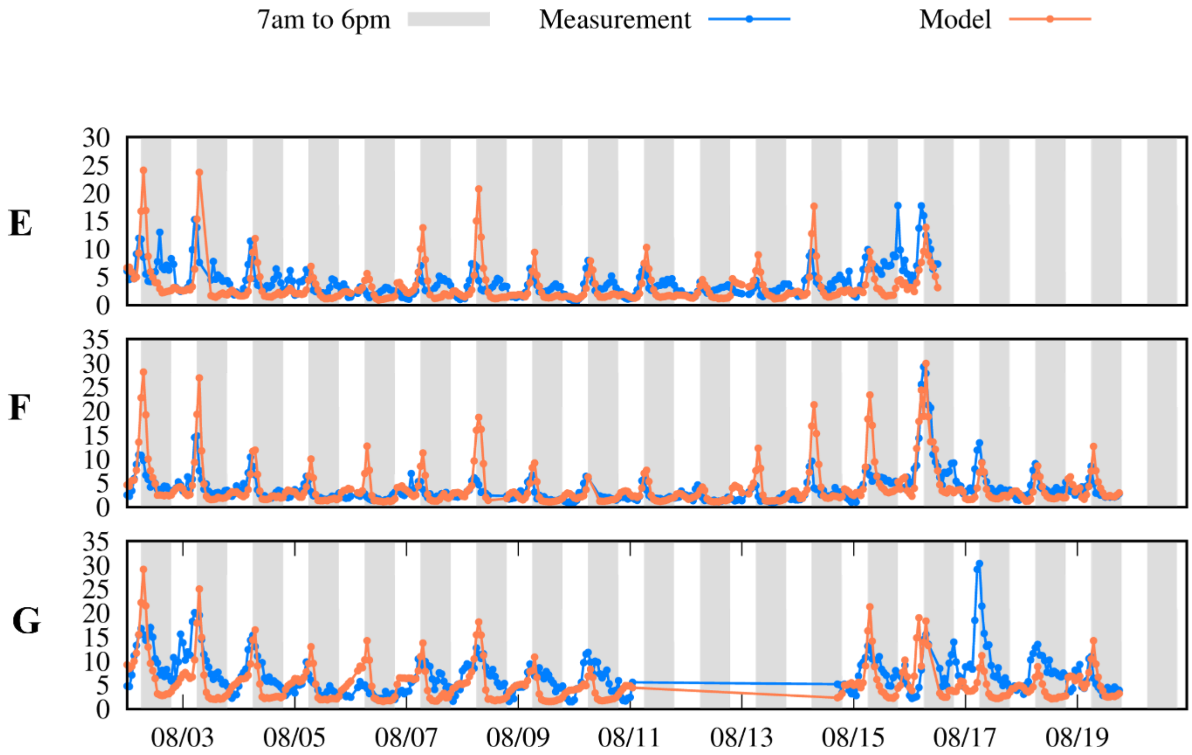

159 **Figure S11.** Measured and modelled August NO_2 concentrations at three representative

160 monitoring sites in Houston.

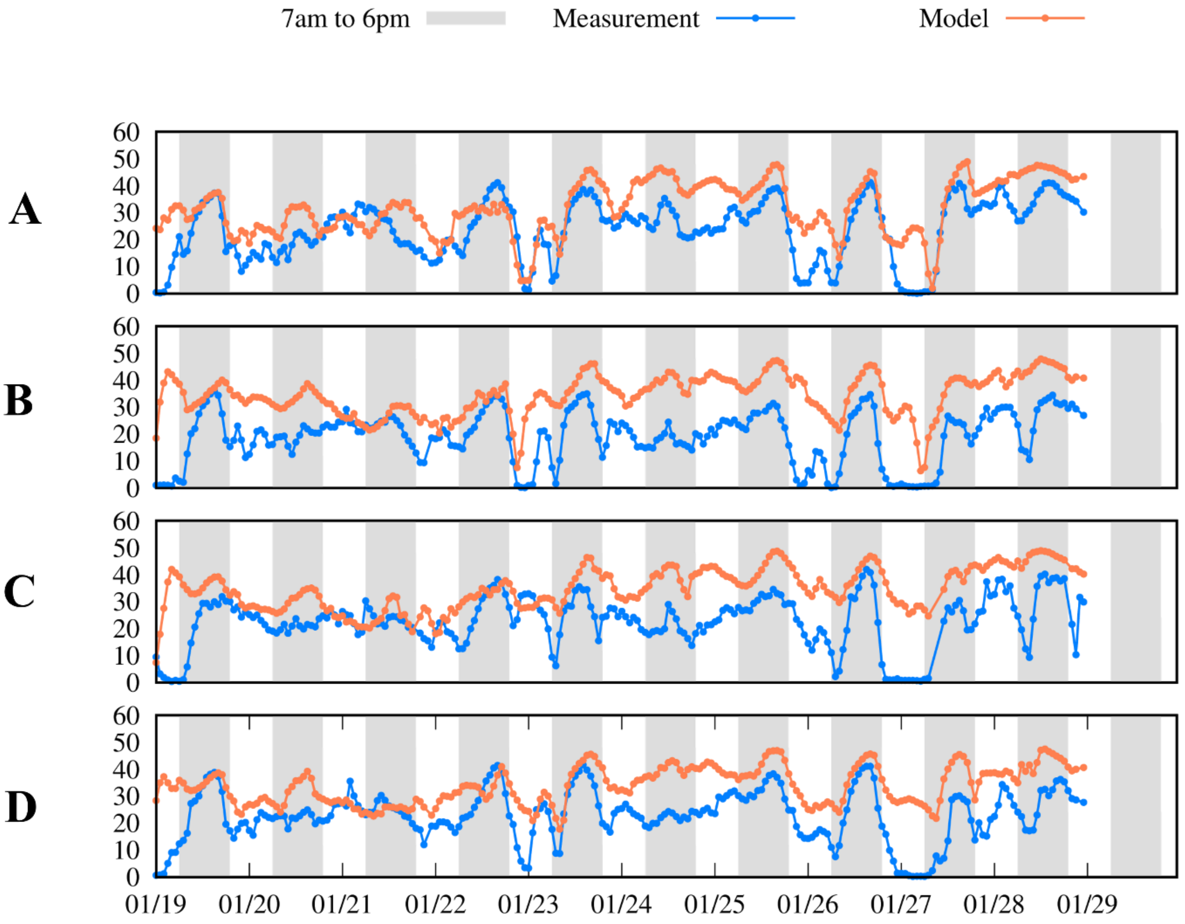

Figure S12. Measured and modelled January O_3 concentrations at four representative monitoring sites in Houston.

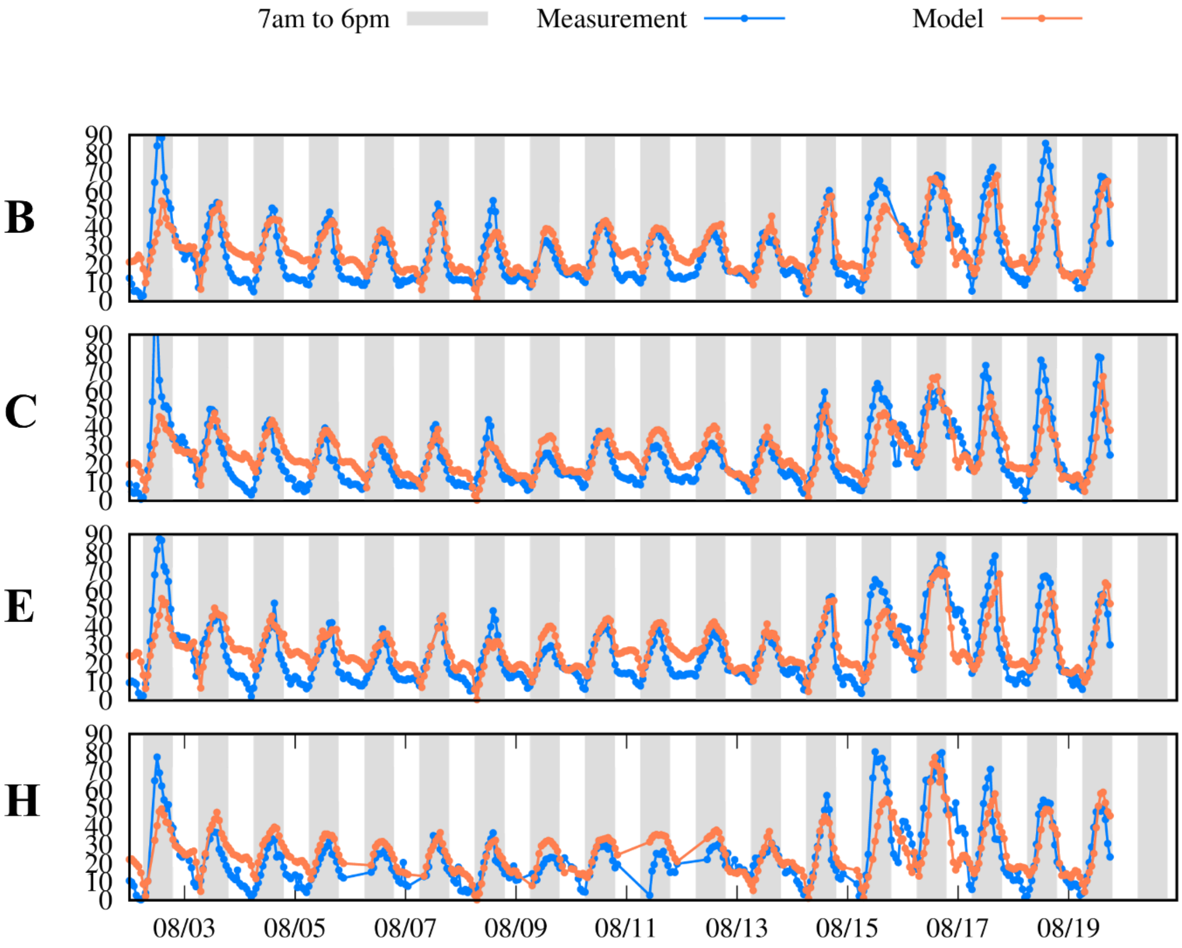

Figure S13. Measured and modelled August O_3 concentrations at four representative monitoring sites in Houston.

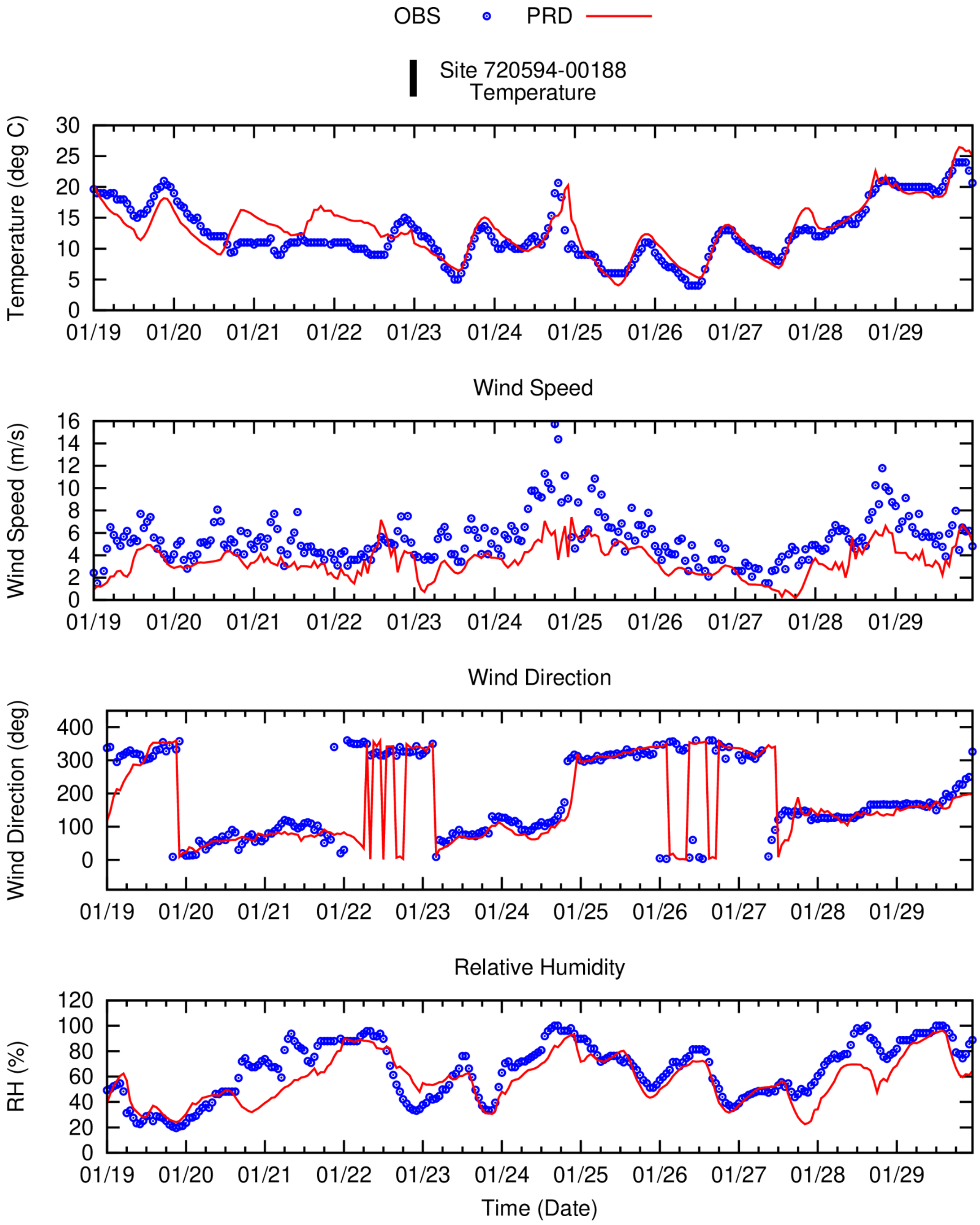

Figure S14. Measured and modelled January surface meteorological parameters at an urban site in Houston.

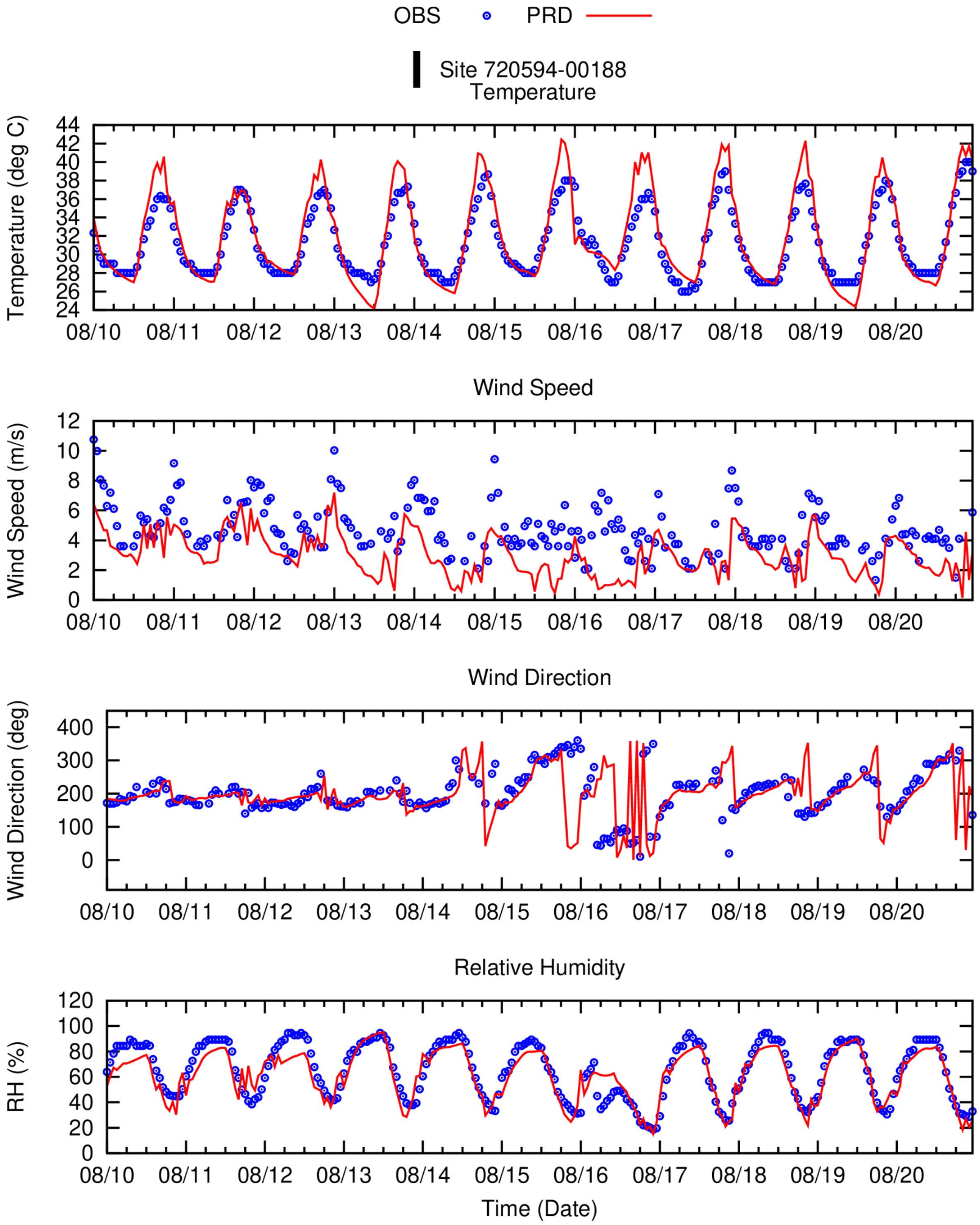

Figure S15. Measured and modelled August surface meteorological parameters at an urban site in Houston.

Table S5. Meteorology evaluation statistics for January and August 2023 for all available National Oceanic and Atmospheric Administration (NOAA) stations in the model domain.

		January	August
TEMP (℃)	MB	-0.16	0.01
	GE	2.19	1.48
	RMSE	2.71	1.89
	OBS	12.64	31.40
	PRD	12.48	31.41
WS (m/s)	MB	-0.88	-0.30
	GE	1.49	1.31
	RMSE	1.93	1.67
	OBS	4.61	4.05
	PRD	4.53	3.75
WD(°)	MB	1.79	-7.25
	GE	26.82	29.39
	RMSE	38.79	44.54
	OBS	170.90	180.48
	PRD	162.04	184.01
RH(%)	MB	-5.98	-4.59
	GE	11.91	8.87
	RMSE	15.53	11.42
	OBS	72.72	65.91
	PRD	66.74	61.32

*TEMP: temperature, WS: windspeed, WD: wind direction, RH: relative humidity; OBS: observation; PRD: prediction; MB: mean bias; GE: gross error; RMSE: root mean square error.

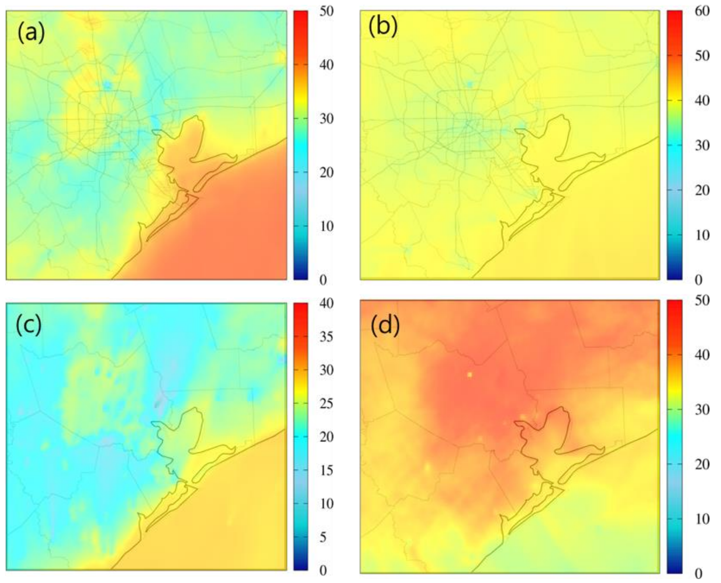

Figure S16. Predicted episode average O_3 concentrations in the Houston area at midnight (a,c) and noon time (b,d) for January (a,b) and August (c,d). The units are ppb.

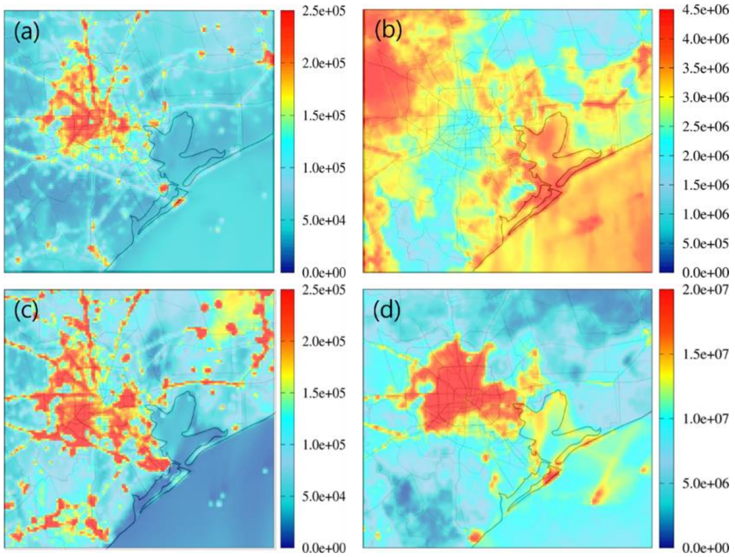

Figure S17. Predicted episode average OH concentrations in the Houston area at midnight (a,c) and noon time (b,d) for January (a,b) and August (c,d). Units are molecules cm^{-3}.

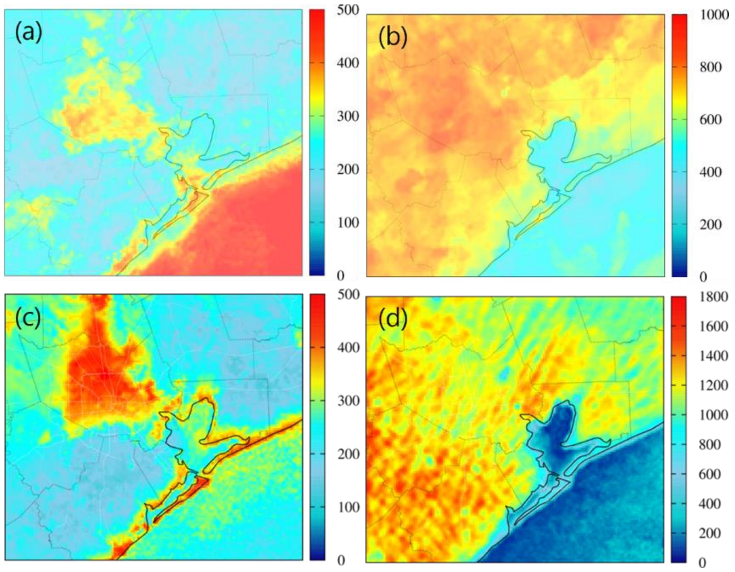

Figure S18. Predicted episode average Planetary Boundary Layer (PBL) height in the Houston area at midnight (a,c) and noon time (b,d) for January (a,b) and August (c,d). The units are meters.

S3.4 Concentration Reported by the Mobile Measurement

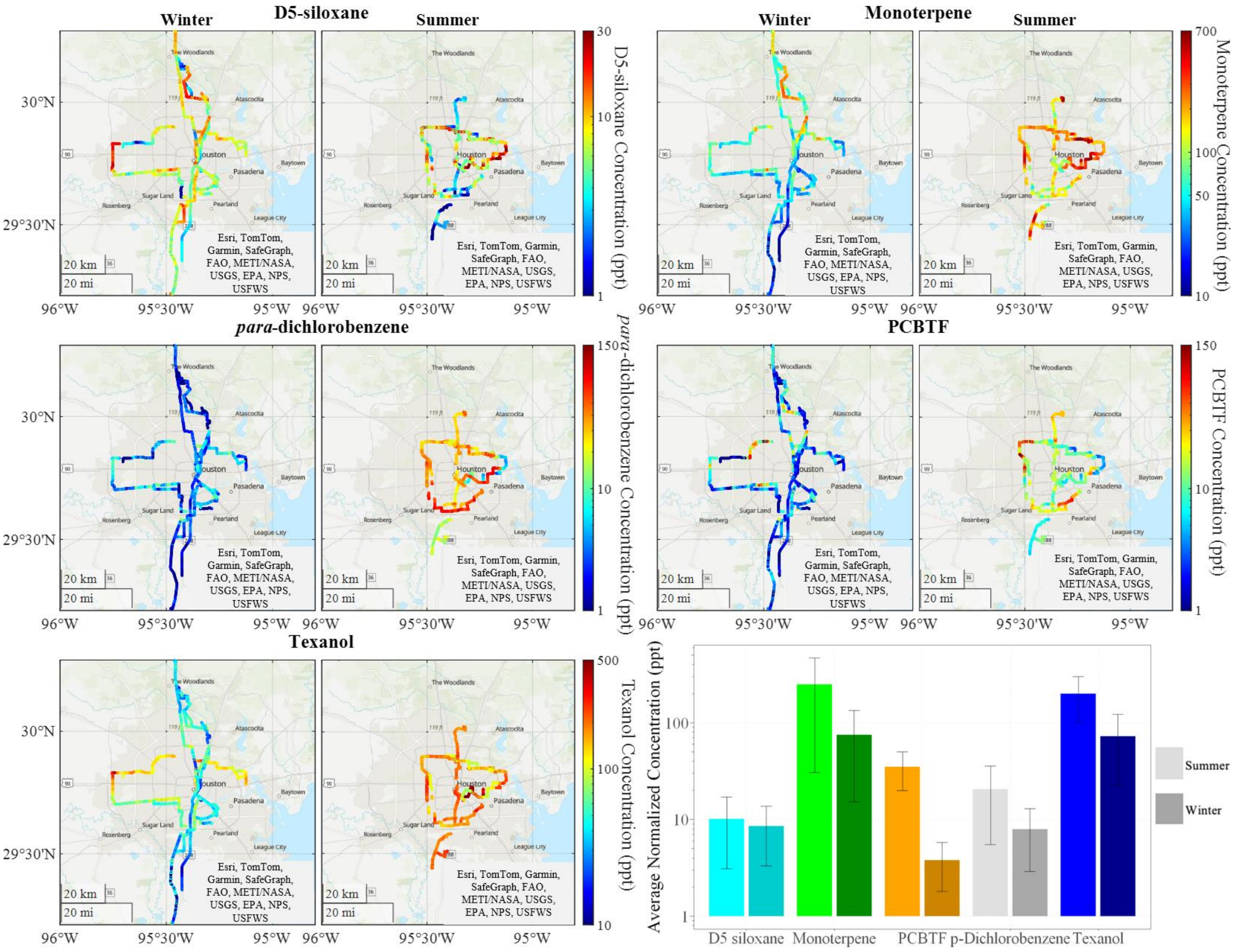

Figure S19. Normalized average winter and summer ambient concentrations and spatial distribution for D5-siloxane, monoterpene, *p*-dichlorobenzene, PCBTF, and Texanol. Note that the scales vary between compounds.

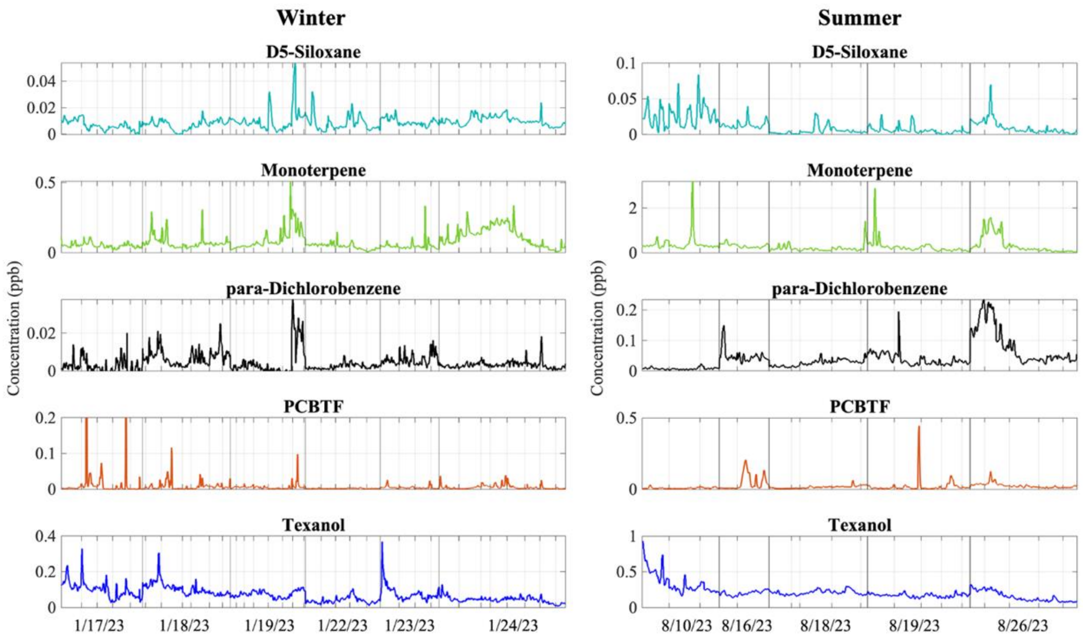

Figure S20. Time series of ambient concentrations for D5-siloxane, monoterpene, *p*-dichlorobenzene, PCBTF, and Texanol. Time series are obtained by 10-second averages, removing points below the LOD and outliers. Thick black lines mark the different sampling days, and gray ticks mark the hours of each day. Note that the scales vary between seasons and compounds.

S3.5 VCP Emissions and Comparisons

The emission and concertation of VCPs derived from this study, and comparison with previous studies conducted in temperature climate zones, are shown in Tables S5-S7.

Table S6: Emission rates for VCP compounds.

**Emission using C_{norm}**					
	**Monoterpene**	**D5-siloxane**	**PCBTF**	***p*-Dichlorobenzene**	**Texanol**
**Winter emission (mg $person^{-1}day^{-1}$)**	334.5 ± 160.3	358.3 ± 209.3	13.0 ± 11.8	15.1 ± 8.5	98.6 ± 20.9
**Summer emission (mg $person^{-1}day^{-1}$)**	1561.7 ± 952.2	620.4 ± 518.5	59.2 ± 37.3	186.1 ± 124.4	296.7 ± 56.3
**Emission using C**					
**Winter emission (mg $person^{-1}day^{-1}$)**	376.8 ± 197.9	382.2 ± 227.5	12.5 ± 11.4	15.1 ± 8.7	101.9 ± 18.4
**Summer emission (mg $person^{-1}day^{-1}$)**	1771.6 ± 1056.1	631.3 ± 513.4	58.3 ± 35.1	210.4 ± 148.2	313.7 ± 62.1

Table S7: VCP concentration comparisons from different locations.

**Compound**	**Season**	**This work (ppt)**	**Previous work (ppt)**
D5-Siloxane	Winter	8.5 ± 5.2	4.5[19]
	Summer	10.1 ± 11.3	125[20]
Monoterpene	Winter	74.9 ± 59.6	75[17]
	Summer	249.6 ± 219.0	170[20]
PCBTF	Winter	7.9 ± 32.2	0-100[21]
	Summer	20.5 ± 35.2	13[20]
p-Dichlorobenzene	Winter	3.8 ± 3.3	
	Summer	34.9 ± 22.6	11[20]
Texanol	Winter	72.4 ± 50.0	0-20,[21] 0.7 - 49.5[22]
	Summer	200.1 ± 99.2	18,[20] 0.7 - 49.5[22]

Table S8: VCP emission comparisons from different locations and various emission calculation methodologies. Note that the emission estimates from Gkatzelis et al.[20] were estimated from their reported emission in kg day,$^{-1}$ using an estimated population desnity of Manhattan as 28,150 people km^{-2} and a total area of 59 km^{2}.[23]

Compound	Season	This work (mg person^{-1}day^{-1})	Previous work (mg person^{-1}day^{-1})
D5-siloxane	Winter	358.3 ± 209.3	336 ± 126,[20] 190 (100-420)[24] 4-235[25] (indoor)
	Summer	620.4 ± 518.5	385 ± 96,[20] 310 (170-690)[26]
Monoterpene	Winter	334.5 ± 160.3	277 ± 89,[20] 520-860[27]
	Summer	1561.7 ± 952.2	362 ± 169[20]
PCBTF	Winter	13.0 ± 11.8	59 ± 9,[20] 1[21]
	Summer	59.2 ± 37.3	72 ± 53[20]
p-Dichlorobenzene	Winter	15.1 ± 8.5	97[28] (indoor), 66 ± 36[20]
	Summer	186.1 ± 124.4	400 ± 72[20]
Texanol	Winter	98.6 ± 20.9	51 ± 35,[20] 0.1[21]
	Summer	296.7 ± 56.3	86 ± 24[20]

References

(1) Krechmer, J.; Lopez-Hilfiker, F.; Koss, A.; Hutterli, M.; Stoermer, C.; Deming, B.; Kimmel, J.; Warneke, C.; Holzinger, R.; Jayne, J.; et al. Evaluation of a New Reagent-Ion Source and Focusing Ion-Molecule Reactor for Use in Proton-Transfer-Reaction Mass Spectrometry. *Anal Chem* **2018**, *90* (20), 12011-12018. DOI: 10.1021/acs.analchem.8b02641.

(2) Alton, M. W.; Browne, E. C. Atmospheric Chemistry of Volatile Methyl Siloxanes: Kinetics and Products of Oxidation by OH Radicals and Cl Atoms. *Environ Sci Technol* **2020**, *54* (10), 5992-5999. DOI: 10.1021/acs.est.0c01368.

(3) Montenegro, A.; Ishibashi, J. S. A.; Lam, P.; Li, Z. Kinetics Study of Reactions of α-Pinene and β-Pinene with Hydroxyl Radical at 1–8 Torr and 240–340 K Using the Relative Rate/Discharge Flow/Mass Spectrometry Method. *The Journal of Physical Chemistry A* **2012**, *116* (49), 12096-12103. DOI: 10.1021/jp307718m.

(4) Tillmann, R.; Saathoff, H.; Brauers, T.; Kiendler-Scharr, A.; Mentel, T. F. Temperature dependence of the rate coefficient for the α-pinene reaction with ozone in the range between 243 K and 303 K. *Physical Chemistry Chemical Physics* **2009**, *11* (13), 2323-2328, 10.1039/B813407C. DOI: 10.1039/B813407C.

(5) Chattopadhyay, A.; Bedjanian, Y.; Romanias, M. N.; Eleftheriou, A. D.; Melissas, V. S.; Papadimitriou, V. C.; Burkholder, J. B. OH Radical and Chlorine Atom Kinetics of Substituted Aromatic Compounds: 4-Chlorobenzotrifluoride (p-ClC6H4CF3). *The Journal of Physical Chemistry A* **2022**, *126* (32), 5407-5419. DOI: 10.1021/acs.jpca.2c04455.

(6) Atkinson, R.; Aschmann, S. M.; Winer, A. M.; Pitts, J. N. Atmospheric gas phase loss processes for chlorobenzene, benzotrifluoride, and 4-chlorobenzotrifluoride, and generalization of predictive techniques for atmospheric lifetimes of aromatic compounds. *Archives of Environmental Contamination and Toxicology* **1985**, *14* (4), 417-425. DOI: 10.1007/BF01055527.

(7) Arnt, R. R.; Seila, R. L.; Bufalini, J. J. Determination of room temperature OH rate constants for acetylene, ethylene dichloride, ethylene dibromide, p-dichlorobenzene and carbon disulfide. *JAPCA, International Journal of Air Pollution Control and Waste Management; (USA)* **1989**, *39:4*, Medium: X; Size: Pages: 453 2016-2005-2020. DOI: 10.1080/08940630.1989.10466544.

(8) Wen, N.; Li, M.; Huo, Y.; Zhou, Y.; Jiang, J.; Ma, Y.; Gu, Q.; Xie, J.; He, M. Homogeneous and heterogeneous atmospheric ozonolysis of chlorobenzene:Mechanism, kinetics and ecotoxicity assessment. *Chemosphere* **2023**, *343*, 140303. DOI: https://doi.org/10.1016/j.chemosphere.2023.140303.

(9) Cater, W. P. L. M., I. L. . Evaluation of Atmopheric Impacts of Selected Coatings VOC Emissions. *California Air Resources Board* **2005**, *00-333*.

(10) Appel, K. W.; Pouliot, G. A.; Simon, H.; Sarwar, G.; Pye, H. O. T.; Napelenok, S. L.; Akhtar, F.; Roselle, S. J. Evaluation of dust and trace metal estimates from the Community Multiscale Air Quality (CMAQ) model version 5.0. *Geosci. Model Dev.* **2013**, *6* (4), 883-899. DOI: 10.5194/gmd-6-883-2013.

(11) Byun, D.; Schere, K. L. Review of the Governing Equations, Computational Algorithms, and Other Components of the Models-3 Community Multiscale Air Quality (CMAQ) Modeling System. *Applied Mechanics Reviews* **2006**, *59* (2), 51-77. DOI: 10.1115/1.2128636 (acccessed 3/18/2025).

(12) Foley, K. M.; Roselle, S. J.; Appel, K. W.; Bhave, P. V.; Pleim, J. E.; Otte, T. L.; Mathur, R.; Sarwar, G.; Young, J. O.; Gilliam, R. C.; et al. Incremental testing of the Community Multiscale Air Quality (CMAQ) modeling system version 4.7. *Geosci. Model Dev.* **2010**, *3* (1), 205-226. DOI: 10.5194/gmd-3-205-2010.

(13) Carter, W.; Heo, G. Development of revised SAPRC aromatics mechanisms. *Atmospheric Environment* **2013**, *77*, 404–414. DOI: 10.1016/j.atmosenv.2013.05.021.
(14) Zhang, J.; He, X.; Ding, X.; Yu, J. Z.; Ying, Q. Modeling Secondary Organic Aerosol Tracers and Tracer-to-SOA Ratios for Monoterpenes and Sesquiterpenes Using a Chemical Transport Model. *Environ Sci Technol* **2022**, *56* (2), 804-813. DOI: 10.1021/acs.est.1c06373.
(15) Agency, U. S. E. P. *2017 Emissions Modeling Platform*. 2017. https://www.epa.gov/air-emissions-modeling/2017-emissions-modeling-platform (accessed August 12, 2023).
(16) Guenther, A.; Karl, T.; Harley, P.; Wiedinmyer, C.; Palmer, P. I.; Geron, C. Estimates of global terrestrial isoprene emissions using MEGAN (Model of Emissions of Gases and Aerosols from Nature). *Atmos. Chem. Phys.* **2006**, *6* (11), 3181-3210. DOI: 10.5194/acp-6-3181-2006.
(17) Zhang, H.; Chen, G.; Hu, J.; Chen, S. H.; Wiedinmyer, C.; Kleeman, M.; Ying, Q. Evaluation of a seven-year air quality simulation using the Weather Research and Forecasting (WRF)/Community Multiscale Air Quality (CMAQ) models in the eastern United States. *Sci Total Environ* **2014**, *473-474*, 275-285. DOI: 10.1016/j.scitotenv.2013.11.121.
(18) Hu, J.; Chen, J.; Ying, Q.; Zhang, H. One-year simulation of ozone and particulate matter in China using WRF/CMAQ modeling system. *Atmos. Chem. Phys.* **2016**, *16* (16), 10333-10350. DOI: 10.5194/acp-16-10333-2016.
(19) Coggon, M. M.; McDonald, B. C.; Vlasenko, A.; Veres, P. R.; Bernard, F.; Koss, A. R.; Yuan, B.; Gilman, J. B.; Peischl, J.; Aikin, K. C.; et al. Diurnal Variability and Emission Pattern of Decamethylcyclopentasiloxane (D5) from the Application of Personal Care Products in Two North American Cities. *Environmental Science & Technology* **2018**, *52* (10), 5610-5618. DOI: 10.1021/acs.est.8b00506.
(20) Gkatzelis, G. I.; Coggon, M. M.; McDonald, B. C.; Peischl, J.; Aikin, K. C.; Gilman, J. B.; Trainer, M.; Warneke, C. Identifying Volatile Chemical Product Tracer Compounds in U.S. Cities. *Environ Sci Technol* **2021**, *55* (1), 188-199. DOI: 10.1021/acs.est.0c05467.
(21) Stockwell, C. E.; Coggon, M. M.; Gkatzelis, G. I.; Ortega, J.; McDonald, B. C.; Peischl, J.; Aikin, K.; Gilman, J. B.; Trainer, M.; Warneke, C. Volatile organic compound emissions from solvent- and water-borne coatings – compositional differences and tracer compound identifications. *Atmos. Chem. Phys.* **2021**, *21* (8), 6005-6022. DOI: 10.5194/acp-21-6005-2021.
(22) Goliff, W. S.; Fitz, D. R.; Cocker, K.; Bumiller, K.; Bufalino, C.; Switzer, D. Ambient measurements of 2,2,4-trimethyl, 1,3-pentanediol monoisobutyrate in Southern California. *Journal of the Air & Waste Management Association* **2012**, *62* (6), 680-685. DOI: 10.1080/10962247.2012.666223.
(23) *The 200 Largest Cities in the United States by Population 2024*. 2024. https://worldpopulationreview.com/us-cities (accessed 2024.
(24) Buser, A. M.; Bogdal, C.; MacLeod, M.; Scheringer, M. Emissions of decamethylcyclopentasiloxane from Chicago. *Chemosphere* **2014**, *107*, 473-475. DOI: 10.1016/j.chemosphere.2013.12.034.
(25) Tang, X.; Misztal, P. K.; Nazaroff, W. W.; Goldstein, A. H. Siloxanes Are the Most Abundant Volatile Organic Compound Emitted from Engineering Students in a Classroom. *Environmental Science & Technology Letters* **2015**, *2* (11), 303-307. DOI: 10.1021/acs.estlett.5b00256.
(26) Buser, A. M.; Kierkegaard, A.; Bogdal, C.; MacLeod, M.; Scheringer, M.; Hungerbühler, K. Concentrations in Ambient Air and Emissions of Cyclic Volatile Methylsiloxanes in Zurich, Switzerland. *Environmental Science & Technology* **2013**, *47* (13), 7045-7051. DOI: 10.1021/es3046586.

(27) Coggon, M. M.; Gkatzelis, G. I.; McDonald, B. C.; Gilman, J. B.; Schwantes, R. H.; Abuhassan, N.; Aikin, K. C.; Arend, M. F.; Berkoff, T. A.; Brown, S. S.; et al. Volatile chemical product emissions enhance ozone and modulate urban chemistry. *Proc Natl Acad Sci U S A* **2021**, *118* (32), e2026653118. DOI: 10.1073/pnas.2026653118 PubMed.
(28) Nazaroff, W.; Weschler Charles, J.; Little John, C.; Hubal Elaine, A. C. Intake to Production Ratio: A Measure of Exposure Intimacy for Manufactured Chemicals. *Environmental Health Perspectives* **2012**, *120* (12), 1678-1683. DOI: 10.1289/ehp.1204992.
